# Handedness, Earnings, Ability and Personality. Evidence from the Lab

**DOI:** 10.1371/journal.pone.0164412

**Published:** 2016-10-27

**Authors:** Marcello Sartarelli

**Affiliations:** Universidad de Alicante, Departamento de Fundamentos del Análisis Económico, Alicante, Spain; Middlesex University, UNITED KINGDOM

## Abstract

Evidence showing that on average left-handed (L), who are 10% in a population, tend to earn less than others is solely based on survey data. This paper is the first to test the relationship between handedness and earnings experimentally and also to assess whether the mechanism underlying it is predominantly cognitive or psychological. Data on 432 undergraduate students show that L do not obtain significantly different payoffs, a proxy for earnings, in a stylised labour market with multiple principals and agents. Similarly, scores in the Cognitive Reflection Test are not significantly different. Data on personality, measured using the Big Five test, show, instead, that L are significantly more agreeable and L females more extroverted. In addition, earnings significantly vary with personality only for L, increasing with extraversion and decreasing with neuroticism. Overall, our results fail to reject the null hypothesis that earnings do not differ by handedness and suggest differences in personality as a novel mechanism to rationalise L’s behaviour.

## Introduction

Survey evidence on young adults in the UK and in the US shows that L earn 10% less, are more frequently in low-skill jobs, with little gender difference, and, in addition, have lower cognitive skills [1]. Comparable results on young adults in the US, although slightly older than in the previous study, show that L males with college education obtain 15% higher wages while no difference is observed for females [2].

In addition, marked gender differences are found among young adults in the UK, with L females earning about 8% less while L men earning 8% more [3]. Overall, this evidence seems to suggest that L are on average somewhat worse off in the labour market. However, estimates may be biased as potentially relevant factors in labour market interactions, such as ability and personality, tend not to be measured in surveys and, hence, cannot be accounted for.

This paper is the first to test whether the hypothesis that L obtain lower earnings (*H*_1_), which found support using survey data, also holds experimentally, i.e. in a controlled environment. In addition, it assesses whether this result is driven by cognitive differences, psychological ones, or by both (*H*_2_).

Testing whether hypothesis *H*_1_ is confirmed when using experimental data on undergraduate students, i.e. young adults, contributes to non-experimental and experimental studies on the consequences of handedness. In addition, hypothesis *H*_2_, by stating that cognitive ability and personality are relevant mechanisms to rationalise L’s behaviour, contributes to recent experimental studies on the relationship between individuals’ cognitive ability, personality and decisions.

Studies in medical science show that L tend to exhibit better information processing and communication thanks to a thicker corpus callosum, a bundle of neural fibers connecting the brain left and right hemispheres [4–6]. However, negligible differences in brain hemispheres asymmetry in grey and white matter [7], as well as in twins by zygosity [8, 9], suggest that the evidence on the determinants of handedness is not conclusive. Similarly, results on cognitive ability are mixed. Some studies show higher ability [10] while others lower one [1, 11], with similar findings for children.

We test the hypotheses set out by borrowing the experimental dataset in Ponti et al. (2016) [Unpublished], with 432 subjects, as they interact in a principal-agent setting capturing some important characteristics of real-life labour markets. In the experiment agents’ earnings depend on whether they put costly effort while principals earn residual profits that increase with the number of agents working for them and putting effort. In addition, the data contain information from the debriefing questionnaire on cognitive ability, measured using the Cognitive Reflection Test (CRT) in [12], and on personality traits from the Big Five [13]. Experiments were run at the Universidad de Alicante Laboratory of Theoretical and Experimental Economics between 2013 and 2015. Overall, our data sample contains 35 L individuals, i.e. 8%.

We find that payoffs, our proxy for labour market earnings, are not significantly different for L (*H*_1_) while they are significantly higher if CRT>0, i.e. above the CRT median value. When we look at performance in the CRT, we also find no significant difference by handedness. Evidence on personality traits from the Big Five shows, instead, that L are significantly more agreeable, and L females are weakly significantly more extroverted. In addition, only for L earnings significantly increase with extraversion, weakly significantly increase with conscientiousness and, in contrast, significantly decrease with neuroticism (*H*_2_).

By finding no support for the hypothesis that L earn less (*H*_1_) in the lab and, in addition, that personality differences explain part of L’s variation in earnings (*H*_2_) our main contribution to the literature is putting forward psychological traits as a mechanism to rationalise L’s behaviour. Our results highlight the importance of collecting information on handedness and personality. The first is an easy-to-measure proxy for personality traits related to social interactions while personality traits are used in the literature as proxies for non-cognitive ability and have been found to explain variation in individuals’ decisions in several contexts.

The structure of the rest of the paper is as follows. The next section reviews the related literature. The following two sections describe respectively the experimental design and data and the results of the empirical analysis. The final section discusses the results and concludes. Additional results are available in S1 Appendix.

## Literature Review

The percentage of L gradually increased from about 2% in 1900 to about 10% in 2000 in developed economies, it is lower in developing ones since L still tend to be forced to use the right hand [14, 15] while, in contrast, it is slightly higher for men, for twins [9] and for prematurely born children [16]. In addition, studies testing consistency in hand use found negligible differences by handedness in primary actions, such as writing, throwing, matching and toothbrush [17].

[1] conducted a study on the relationship between handedness and several policy-relevant outcomes. Representative survey data on about 40,000 young adults, with age ranging from 20 to mid-30s, were obtained from multiple sources: the UK (National Child Development Study and the British Cohort Study) and the US (Children and Young Adults and National Longitudinal Survey of Youth). The results show that L earn 10-12% less, that is approximately equal to the return of an extra year of schooling in the data used. In addition, they are about 10-20% less frequently in high-skill jobs, with little gender difference, have lower cognitive skills by about 10% and the frequency of behavioral and learning problems is 10-30% higher.

By using similar outcomes, related studies tested whether the relationship between handedness and earnings varies by education and by gender. Evidence on about 2,000 young adults in the US (National Longitudinal Survey of Youth) aged 28-35, i.e. on average slightly older than those in the data sample in [1], shows that L males with college education obtain 15% higher wages [2]. In contrast, no significant difference is observed for females. Results obtained using survey data on about 5,000 young adults in the UK (National Child Development Survey) also show gender differences, with L females earning about 8% less than other females and, instead, L males earning 8% more than other males [3].

Results showing that L are over-represented among talented and creative individuals [10, 18] are based on a small number of observations and are overall little robust, as shown in [1], while over-representation among low ability individuals is more robust [1, 19]. Related evidence on differences in cognitive ability by handedness is mixed. Some studies show higher ability for L [10] while others lower [1, 11].

When looking at differences in children’s ability by handedness, results are also mixed. Being L is associated with better school performance and leadership skills for boys and worse ones for girls in a survey of 1,700 primary school children in France [20] and in a longitudinal study of about 5,000 children aged 4-5 in Australia [21]. In contrast, it is also associated with worse learning, cognitive, social and language skills, driven by boys, for about 6,000 children aged about 13 in a large and representative survey (National Longitudinal Survey of Youth) in the US [22]. Earlier studies also show mixed evidence, some finding that L children have lower ability [23, 24] while others higher [25] or no significant difference [26]. Overall, mixed results on differences in ability by handedness for both adults and children show that the relationship between handedness and ability is age-invariant. This suggests that different learning trajectories, from childhood to adulthood, by handedness are not a relevant factor to explain the differences observed in the literature.

Physiological differences correlated with handedness have been detected in medical science, with L showing a wider corpus callosum (CC). This is a 10cm long and flat bundle of neural fibers located below the brain cortex that, by connecting the brain left and right hemispheres, is responsible for information processing and communication between them. Differences in CC by handedness seem to be greater for men [27]. No difference in CC size is observed, instead, in adults relative to children, while CC size slightly decreases in old age [28].

Differences in genes that are weakly linked to neuro-developmental disorders [29–32] and, in addition, in prenatal thumb sucking driven by males [33] seem to support the hypothesis that handedness is partially due to genetics. However, results showing that several genes, rather than just one, can explain this relationship [31, 34] suggest that the evidence is not conclusive.

Handedness has also been widely studied in psychology. Differences by handedness in neural activity associated with motivation, that are observed in experiments [35, 36], seem to suggest a complex relationship between handedness, genetic factors and behavioural ones. Related studies show that L have higher self-reported behavioral inhibition [37, 38], with higher inhibition for L females [39] and longer latency before starting a task [40, 41]. Finally, L show initially higher anxiety due to task novelty [42] while it subsequently decreases with the number of tasks [43].

## Materials and Methods

We test experimentally the hypotheses set out in this paper by borrowing the relevant variables from a rich dataset on 432 subjects. It was obtained to study in Ponti et al. (2016) [Unpublished] the determinants and monetary consequences of endogenising principals selection thanks to a laboratory experiment with multiple principals and agents, i.e. a stylised labour market. From phase 3 in Ponti et al. (2016) [Unpublished] we use data on earnings in the labour market. We also use data from the debriefing questionnaire administered at the end of the experiment. Students from the Universidad de Alicante were recruited to participate in the experiment by using ORSEE [44]. Experimental sessions were carried out at the Laboratory of Theoretical and Experimental Economics (LaTEx), using z-Tree [45], in 2013, 2014 and 2015.

Approval for the experiment was given by the LaTEx Ethics Committee. Participants gave their consent to participate in social experiments when they signed up in ORSEE. When, before the experiment started, instructions about its content were read aloud to all participants, they were informed that they could leave the experiment at any stage. Two separate approvals were obtained: the first one to run the experiment that generated the full dataset used in Ponti et al. (2016) [Unpublished] and the second one to use a subset of the dataset in Ponti et al. (2016) [Unpublished] to study the relationship between handedness and earnings.

The stylised labour market in phase 3 in Ponti et al. (2016) [Unpublished] works as follows. Firstly, subjects chose whether being agents or principals. Secondly, agents were randomly paired and principals competed to hire them by offering payoffs for pairs of agents that depend on their effort in the form of 2x2 effort games. Finally, both agents in each pair chose a contract among those offered by principals and then whether to put effort in the randomly chosen contract between the two that were selected in a pair. Principals’ payoffs increase in the number of agents pairs hired and putting effort, while agents’ payoffs are higher if at least one in a pair, or both, put effort. At the beginning of each experimental session 24 players were randomly divided in 2 groups of 12 players, with no interaction between groups throughout the session. In this phase there were 4 principals and 8 agents in each group and the phase consisted of 24 identical rounds, with principals being selected at the beginning of each round.

There are two additional phases in Ponti et al. (2016) [Unpublished], that are briefly described for completeness although data generated from them are not used in this paper. In phase 1 all subjects were paired randomly for 24 rounds and each player in a pair chose one out of four payoff pairs, with one payoff for each player in a pair, in a random dictator game-type protocol. Evidence from this phase is used to estimate subjects’ social preferences. In phase 2, similarly to phase 1, subjects were paired randomly for 24 rounds. In this phase, firstly each player in a pair chose one out of four 2x2 effort games; secondly, a randomly drawn game between the chosen ones in a pair was played. Evidence from this phase is used to estimate reciprocity preferences and beliefs over the effort game.

In some experimental sessions subjects paid nothing to be principals, to mimic free entry. In other sessions, instead, principals were selected by way of a second price auction, i.e. costly entry, with subjects’ bids being paid by an initial endowment they received, to avoid losses. If more than 4 subjects per group were willing to be principals, 4 principals were drawn randomly among them. In the event of fewer than 4 subjects, instead, as many subjects as were necessary to obtain 4 principals were drawn randomly from the pool of subjects not willing to be principals. Evidence from Ponti et al. (2016) [Unpublished] shows that an excess of subjects willing to be principals is substantially more frequent than a deficit.

All monetary payoffs in the experiment were expressed in Spanish pesetas with an exchange rate of 166 pesetas for 1 euro. The final payoffs for each subject were calculated as the sum of the payoffs in all phases in the Ponti et al. (2016) [Unpublished] experiment. Subjects earned on average 20 euros for an experimental session that lasted, all included, about 2 hours. Additional information about the experimental design is available in section 2 in Ponti et al. (2016) [Unpublished] and also in [46], the study from which the experimental design was borrowed.

It is standard practice, for all experiments at LaTEx, to use Spanish pesetas as experimental currency. The reason for this design choice is twofold. First, it mitigates integer problems, compared with, for example, US dollars or euros. Although Spanish pesetas were replaced by euros in 2002, they are still used to express monetary values in everyday life with, for example, several supermarkets displaying prices in both currencies. Second, by using a real, as opposed to an artificial currency, we avoid the problem of framing the incentive structure of the experiment, which arises when using a scale with no cognitive content.

From the debriefing questionnaire we use a dummy equal to 1 if a subject is left-handed (L) and a dummy equal to 1 for females. We also use two measures of cognitive ability. The first is the CRT score [12], that takes as possible values integers in the interval from 0, when a subject does not answer correctly any question in the CRT, to 3, when all three questions are answered correctly. The second is a dummy equal to 1 if the CRT score is greater than 0, the median value in our data. Since the support of the CRT score is integers in a very small interval, using in regressions a dummy to measure if the CRT score is greater than the median value simplifies results interpretation.

The main advantage of using the CRT is that it is short and accessible to any individual who learnt the basic algebra taught in compulsory education. Perhaps one of its main disadvantages, instead, is lacking questions that test logic ability. However, evidence in [47] and references therein show that ability in logic and in math tend to be positively correlated. The order in which the 3 CRT questions were presented is the same for all subjects and in all experimental sessions, as in [12]. In addition, the test was not incentivised since it was part of the debriefing questionnaire.

In section A in S1 Appendix we also test whether L differ in the GPA over all exams taken by students in the degree studied at the Universidad de Alicante by the time of the experiment, as it is a proxy for both cognitive and non-cognitive ability. Tests of whether L differ in preferences towards risk are instead performed in the working paper version [48]. However, the number of observations for the lottery choices used to elicit risk preferences is lower than for payoffs in the stylised labour market.

Finally, we use data from a reduced version of the Big Five test (BF) to obtain measures of the following personality traits: agreeableness, conscientiousness, extraversion, neuroticism and openness. The BF test consists of a set of questions on different aspects of these traits. Answers to each question take as possible values integers between 1 and 7, indicating that a subject believes that her/his personality is respectively very poorly or very well described by the aspect of a trait. Variables measuring traits are constructed as the mean over the answers to the set of questions on a trait and, by construction, take as values integers or decimals in the same interval [13, 49]. The list of questions for each personality trait are shown in Table F in S1 Appendix.

We also use the BF short version [50]. It was designed to capture the following aspects of personality traits, that are frequently used in research in psychology as they seem to have good explanatory power across studies: find faults with others, be generally trusting (agreeableness); do a thorough job, tend to be lazy (conscientiousness); be reserved, be outgoing and sociable (extraversion); be relaxed and handle stress well, get nervous easily (neuroticism); have an active imagination, have few artistic interests (openness).

Summary statistics of the variables used in the empirical analysis are shown in Table 1. 8% of individuals are L in the data, a figure not substantially different from 10% in related studies based on survey data, and Table A in S1 Appendix shows that the number of L individuals in the data is 35. Table 1 also shows that the data are close to being gender-balanced, with 48% females. The mean of the CRT score shows that on average subjects answer correctly fewer than one out of the three questions in the CRT and that 0 is the median value. When looking at the mean value of the dummy *I*(*CRT* > 0), it shows that the percentage of subjects with at least one right answer is about 40%.

**Table 1:** Summary statistics.

	Mean	Median	Std. dev.	Min	Max	N. obs.s
Left-handed	0.081	.	0.273	0.000	1.000	432
Female	0.479	.	0.500	0.000	1.000	432
CRT score	0.699	0.000	0.986	0.000	3.000	432
*I*(*CRT* > 0)	0.396	.	0.490	0.000	1.000	432
Experiment payoff (euros)	6.434	6.445	4.938	0.000	19.887	432
*Big Five personality traits*						
AGReeableness	4.852	4.800	0.645	2.400	6.400	432
CONscientiousness	5.418	5.600	0.903	2.000	7.200	432
EXTraversion	4.619	4.600	1.282	1.200	7.000	432
NEUroticism	3.931	3.833	1.201	1.000	7.000	432
OPEnness	5.482	5.571	0.849	2.143	7.143	432
SHOrt (10 questions)	4.756	4.800	0.656	2.600	6.300	432

In addition, Table 1 shows that the mean payoff in the stylised labour market in Ponti et al. (2016) [Unpublished], it is about 6.4 euros with a standard deviation of about 5 euros. The bottom part of the table shows summary statistics of personality traits in the BF. Their mean values range approximately between 3.9 for neuroticism and 5.48 for openness and are in line with median values, suggesting that the distributions tend to be centered at the mean. Their standard deviations also vary, between 0.65 for agreeableness and 1.28 for extraversion. Additional results in Table A in S1 Appendix show in Panel A that the percentage by gender of L is similar to the figure for the full data sample and the same holds for the percentage of subjects with CRT>0. The same table also shows in Panel B that 22.6% of subjects answer correctly 2 or more questions in the CRT and only 7.6% all 3 questions.

Table 2 shows correlations between outcomes and explanatory variables used in the empirical analysis. The first column on the left-hand side shows that no variable is significantly correlated with being L. The absence of significant correlations between L and other predetermined characteristics used as explanatory variables in earnings regressions simplifies the interpretation of regression estimates. With a low percentage of L and, on top of it, fewer observations in experimental relative to non-experimental data, this applies in particular to coefficients of interactions, for example, between the L dummy and gender or cognitive ability dummies.

**Table 2:** Correlations.

	L	F	CRT	*I*(*CRT* > 0)	Exp.payoff	Big Five (BF) personality traits					
						AGR	CON	EXT	NEU	OPE	SHO
Left-handed (L)	1.000										
Female (F)	0.004	1.000									
CRT score	-0.030	-0.220***	1.000								
*I*(*CRT* > 0)	-0.015	-0.236***	0.877***	1.000							
Experiment payoff	0.031	-0.008	0.091*	0.164***	1.000						
BF AGReeableness	0.068	0.066	-0.023	-0.069	0.034	1.000					
BF CONscientiousness	0.026	0.095**	-0.072	-0.053	0.091*	0.329***	1.000				
BF EXTraversion	0.045	-0.060	-0.070	-0.067	0.092*	0.150***	0.120**	1.000			
BF NEUroticism	0.000	0.255***	-0.192***	-0.154***	0.023	-0.176***	0.040	-0.017	1.000		
BF OPEnness	0.023	-0.081*	0.124**	0.110**	0.093*	0.210***	0.318***	0.245***	-0.019	1.000	
BF SHOrt (10 questions)	0.052	0.110**	-0.137***	-0.119**	0.185***	0.350***	0.505***	0.483***	0.396***	0.490***	1.000

* *p* < 0.10

** *p* < 0.05

*** *p* < 0.01

The second column shows that females have a highly significantly lower CRT, measured both as the number of right answers and as a dummy equal to 1 if the number of correct answers is greater than 0. This is in line with evidence in the related literature (see for a literature review [51, 52]). Females also score significantly higher in BF conscientiousness and neuroticism while weakly significantly lower in openness. The third and fourth columns show a positive and significant correlation between the CRT and experimental payoffs, with the significance level being higher for the *I*(*CRT* > 0) dummy. CRT is also negatively and significantly correlated with neuroticism and BF short while it is positively correlated with openness.

In addition, Table 2 shows that experimental payoffs are positively but weakly significantly correlated with conscientiousness, extraversion and openness while they are highly significantly correlated with BF short. Finally, the table shows significant correlations between BF traits. For example, agreeableness is positively correlated with conscientiousness, as well with other traits, while it is negatively correlated with neuroticism. Summary statistics of answers to questions in the BF test and their correlations with our outcomes of interest are shown respectively in Tables F and G in S1 Appendix.

## Results

In this section we firstly we describe regression estimates of the relationship between earnings, handedness and ability to assess if earnings are lower for L (*H*_1_). Then, we show comparable estimates obtained after adding to the earnings regressions information on BF personality traits, to assess their relevance in explaining L’s behaviour (*H*_2_). In all regressions, robust standard errors were used.

### Handedness and Cognitive Ability

Table 3 shows estimates of regressions with as outcome the dummy *I*(*CRT* > 0), that is equal to 1 if CRT>0, and as independent variables the dummy equal to 1 for L, a dummy equal to 1 for females (F) and their interaction. Estimates show that the probability that CRT>0 is not significantly different for L and the coefficient is small (7-13% of the mean probability that CRT>0, hereafter mean) while it is significantly lower for females (55-58% mean). In addition, the L effect is not different by gender, as the non-significant coefficient of the interaction between *L and female dummies* shows. Using as outcome the CRT score in an ordered logit regression, as in [52], leads to similar results, as shown in Table B in S1 Appendix.

**Table 3:** Regression of the dummy I(CRT>0).

L	-0.027	-0.025	0.053
	(0.085)	(0.080)	(0.123)
F		-0.231***	-0.218***
		(0.046)	(0.048)
L*F			-0.161
			(0.157)
Constant	0.398***	0.509***	0.502***
	(0.025)	(0.034)	(0.035)
Observations	432	432	432

* *p* < 0.10

** *p* < 0.05

*** *p* < 0.01

Table 4 shows estimates of regressions using as outcome payoffs, measured in euros, in the stylised labour market experiment in Ponti et al. (2016) [Unpublished]. Although payoffs are slightly higher for L (7-12% of a standard deviation, hereafter s.d.), estimates are not significant. They are, instead, highly significantly higher if CRT>0 (34-54% s.d.). This result is driven by males, as shown by the negative and significant coefficient of the interaction between the *dummies for female and for CRT>0*, as well as by positive and significant coefficients of the *CRT dummy* in subsample estimates for males in the bottom part of the table. Results are unchanged when using standard errors clustered by session and independent subjects groups in a session, to correct them for that part of variation in payoffs that is due to subjects in the same session and group, as shown in Table D in S1 Appendix.

**Table 4:** Regression of labour market payoffs (euros) in Ponti et al. (2016) [Unpublished].

	***Full sample regressions***					
L	0.569		0.613	0.575	0.554	0.340
	(0.823)		(0.811)	(0.993)	(0.991)	(1.601)
CRT		1.652***	1.657***	1.649***	1.722***	2.662***
		(0.485)	(0.485)	(0.509)	(0.516)	(0.685)
L*C				0.101	0.158	-0.602
				(1.713)	(1.706)	(2.370)
F					0.320	1.068*
					(0.477)	(0.624)
L*F						0.257
						(2.036)
F*C						-2.253**
						(1.036)
L*C*F						3.240
						(3.002)
Constant	6.388***	5.780***	5.728***	5.731***	5.549***	5.124***
	(0.249)	(0.296)	(0.306)	(0.312)	(0.406)	(0.461)
Observations	432	432	432	432	432	432
	***Subsample regressions by gender***					
	**Female**			**Male**		
L	1.018	1.079	0.597	0.147	0.008	0.340
	(1.091)	(1.081)	(1.258)	(1.224)	(1.200)	(1.600)
CRT		0.568	0.409		2.614***	2.662***
		(0.739)	(0.777)		(0.654)	(0.685)
L*C			2.638			-0.602
			(1.845)			(2.369)
Constant	6.308***	6.146***	6.191***	6.461***	5.148***	5.124***
	(0.352)	(0.414)	(0.421)	(0.354)	(0.451)	(0.461)
Observations	207	207	207	225	225	225

* *p* < 0.10

** *p* < 0.05

*** *p* < 0.01

### Handedness and Personality

Table 5 shows estimates of regressions using as outcomes BF agreeableness (AGR) and conscientiousness (CON). The panel on the left-hand side shows that AGR is significantly higher for L (25-48% s.d.) and it is driven by females, as shown by subsample estimates by gender in the bottom part of the table. In addition, AGR is significantly lower for L with CRT>0, as shown by the coefficient of the interaction between *L and CRT>0 dummies*, and it is driven by males, as shown by subsample estimates by gender. Estimates on the right-hand side, instead, show that CON is not significantly different by handedness nor by any other predetermined characteristic used as explanatory variables. Each personality trait was obtained as the mean over answers to a set of questions measuring different aspects of a trait. Additional details about the BF test can be found in the Materials and Methods section.

**Table 5:** Regressions of Big Five AGReeableness and CONscientiousness.

	***Full sample regressions***											
	**AGReeableness**						**CONscientiousness**					
L	0.161*		0.158*	0.311***	0.307***	0.309**	0.086		0.083	0.166	0.156	0.281
	(0.091)		(0.089)	(0.099)	(0.099)	(0.130)	(0.155)		(0.154)	(0.174)	(0.176)	(0.244)
I(CRT>0) (C)		-0.090	-0.089	-0.057	-0.043	-0.062		-0.098	-0.097	-0.079	-0.044	-0.123
		(0.064)	(0.064)	(0.068)	(0.070)	(0.091)		(0.090)	(0.090)	(0.094)	(0.096)	(0.130)
L*C				-0.408**	-0.397**	-0.508**				-0.222	-0.194	-0.412
				(0.179)	(0.177)	(0.197)				(0.338)	(0.335)	(0.422)
F					0.063	0.037					0.155*	0.085
					(0.065)	(0.086)					(0.089)	(0.118)
L*F						-0.001						-0.189
						(0.189)						(0.339)
F*C						0.040						0.184
						(0.145)						(0.194)
L*C*F						0.478						0.646
						(0.390)						(0.615)
Constant	4.839***	4.888***	4.875***	4.862***	4.826***	4.841***	5.411***	5.457***	5.450***	5.443***	5.355***	5.394***
	(0.033)	(0.040)	(0.041)	(0.042)	(0.057)	(0.066)	(0.045)	(0.055)	(0.057)	(0.058)	(0.079)	(0.092)
Observations	432	432	432	432	432	432	432	432	432	432	432	432
	***Subsample regressions by gender***											
	**Female**			**Male**			**Female**			**Male**		
L	0.305**	0.302**	0.308**	0.024	0.029	0.309**	0.127	0.135	0.092	0.045	0.054	0.281
	(0.125)	(0.126)	(0.138)	(0.121)	(0.115)	(0.130)	(0.206)	(0.206)	(0.236)	(0.227)	(0.223)	(0.243)
C		-0.024	-0.022		-0.102	-0.062		0.075	0.061		-0.156	-0.123
		(0.108)	(0.113)		(0.085)	(0.091)		(0.138)	(0.144)		(0.124)	(0.130)
L*C			-0.030			-0.508**			0.234			-0.412
			(0.337)			(0.197)			(0.448)			(0.422)
Constant	4.872***	4.878***	4.878***	4.810***	4.861***	4.841***	5.497***	5.475***	5.479***	5.332***	5.411***	5.394***
	(0.048)	(0.054)	(0.055)	(0.045)	(0.064)	(0.066)	(0.063)	(0.073)	(0.074)	(0.065)	(0.090)	(0.092)
Observations	207	207	207	225	225	225	207	207	207	225	225	225

* *p* < 0.10

** *p* < 0.05

*** *p* < 0.01

In addition, estimates in Table 6 show on the left-hand side that extraversion (EXT) is higher for L (4-34% s.d.) although estimates are not significant, while it is weakly significantly lower for females (17-24% s.d.). In addition, subsample estimates by gender show that EXT is weakly significantly higher for L females (40-52% s.d.). Estimates on the right-hand side in Panel B show that neuroticism (NEU) is not significantly different by handedness and point estimates have a mixed sign. It also shows that NEU is significantly lower if CRT>0 (18-32% s.d.). When adding the *female dummy*, instead, the coefficient of the *CRT dummy* loses significance while NEU becomes highly significantly higher for females (45-46% s.d.).

**Table 6:** Regressions of Big Five EXTraversion and NEUroticism.

	***Full sample regressions***											
	**EXTraversion**						**NEUroticism**					
L	0.209		0.205	0.420	0.434	0.046	0.002		-0.008	0.143	0.106	-0.015
	(0.224)		(0.221)	(0.279)	(0.283)	(0.472)	(0.233)		(0.226)	(0.280)	(0.270)	(0.340)
C		-0.175	-0.173	-0.127	-0.176	-0.258		-0.378***	-0.378***	-0.346***	-0.221*	-0.245
		(0.127)	(0.128)	(0.133)	(0.135)	(0.174)		(0.117)	(0.118)	(0.122)	(0.121)	(0.162)
L*C				-0.576	-0.614	-0.217				-0.404	-0.306	-0.055
				(0.443)	(0.446)	(0.638)				(0.468)	(0.465)	(0.577)
F					-0.213*	-0.310*					0.552***	0.534***
					(0.125)	(0.160)					(0.113)	(0.150)
L*F						0.621						0.193
						(0.585)						(0.509)
F*C						0.176						0.057
						(0.275)						(0.246)
L*C*F						-0.620						-0.733
						(0.862)						(0.853)
Constant	4.602***	4.688***	4.671***	4.653***	4.774***	4.829***	3.931***	4.081***	4.082***	4.069***	3.755***	3.765***
	(0.064)	(0.078)	(0.080)	(0.081)	(0.102)	(0.114)	(0.060)	(0.073)	(0.075)	(0.076)	(0.098)	(0.113)
Observations	432	432	432	432	432	432	432	432	432	432	432	432
	***Subsample regressions by gender***											
	**Female**			**Male**			**Female**			**Male**		
L	0.528*	0.513*	0.667*	-0.088	-0.074	0.046	0.058	0.033	0.177	-0.059	-0.046	-0.015
	(0.306)	(0.303)	(0.345)	(0.320)	(0.317)	(0.472)	(0.335)	(0.330)	(0.379)	(0.300)	(0.298)	(0.340)
C		-0.132	-0.082		-0.275	-0.258		-0.235	-0.188		-0.249	-0.245
		(0.204)	(0.213)		(0.167)	(0.174)		(0.178)	(0.185)		(0.155)	(0.162)
L*C			-0.837			-0.217			-0.788			-0.055
			(0.580)			(0.637)			(0.629)			(0.576)
Constant	4.496***	4.533***	4.519***	4.700***	4.838***	4.829***	4.246***	4.312***	4.299***	3.643***	3.768***	3.765***
	(0.095)	(0.111)	(0.113)	(0.087)	(0.112)	(0.114)	(0.083)	(0.097)	(0.098)	(0.081)	(0.111)	(0.113)
Observations	207	207	207	225	225	225	207	207	207	225	225	225

* *p* < 0.10

** *p* < 0.05

*** *p* < 0.01

Table 7 reports the last set of estimates from regressions with personality traits as outcomes. The panel on the left-hand side shows that openness (OPE) is higher for L (8-32% s.d.) although the difference is not significant. OPE is, instead, significantly higher if CRT>0 (13-24% s.d.) for all but one specification, with this result being driven by females, as shown by subsample estimates by gender in the bottom part of the table. Estimates on the right-hand side show that BF short (SHO), obtained using 10 questions on different traits [50], tends to higher for L (19-57% s.d.) although it is only significant once CRT and gender have been accounted for. In addition, SHO tends to be lower if CRT>0 (13-29% s.d.) although this estimate is not significant for all specifications. When looking at the interaction between *L and CRT>0 dummies*, estimates show that SHO is significantly lower for L with CRT>0. The positive relationship between SHO and L tends to be driven by those BF questions used to obtain SHO that are significantly associated with L: find faults with others (AGR) negatively and be outgoing, sociable (EXT) positively, as shown in Tables H and J in S1 Appendix.

**Table 7:** Regressions of Big Five OPEnness and SHOrt.

	***Full sample regressions***											
	**OPEnness**						**SHOrt (10 questions)**					
L	0.071		0.076	0.131	0.138	0.273	0.126		0.122	0.352***	0.345***	0.371***
	(0.118)		(0.119)	(0.149)	(0.147)	(0.224)	(0.122)		(0.116)	(0.126)	(0.127)	(0.106)
C		0.191**	0.192**	0.203**	0.180*	0.111		-0.159**	-0.158**	-0.109	-0.085	-0.190**
		(0.083)	(0.084)	(0.089)	(0.092)	(0.119)		(0.066)	(0.066)	(0.068)	(0.069)	(0.092)
L*C				-0.147	-0.165	-0.161				-0.616***	-0.597***	-0.570**
				(0.245)	(0.241)	(0.307)				(0.229)	(0.230)	(0.226)
F						-0.102	-0.144				0.106*	0.018
						(0.086)	(0.113)				(0.063)	(0.081)
L*F							-0.208					-0.033
							(0.294)					(0.218)
F*C							0.175					0.249*
							(0.190)					(0.138)
L*C*F							-0.333					-0.124
							(0.536)					(0.583)
Constant	5.476***	5.406***	5.400***	5.395***	5.453***	5.477***	4.746***	4.819***	4.809***	4.789***	4.729***	4.779***
	(0.043)	(0.052)	(0.054)	(0.055)	(0.075)	(0.087)	(0.033)	(0.038)	(0.040)	(0.040)	(0.053)	(0.060)
Observations	432	432	432	432	432	432	432	432	432	432	432	432
	***Subsample regressions by gender***											
	**Female**			**Male**			**Female**			**Male**		
L	-0.053	-0.025	0.065	0.189	0.184	0.273	0.210	0.212	0.339*	0.045	0.057	0.371***
	(0.172)	(0.176)	(0.190)	(0.153)	(0.154)	(0.224)	(0.189)	(0.191)	(0.191)	(0.149)	(0.135)	(0.106)
C		0.256*	0.286*		0.098	0.111		0.017	0.058		-0.235***	-0.190**
		(0.141)	(0.147)		(0.112)	(0.119)		(0.104)	(0.103)		(0.086)	(0.092)
L*C			-0.494			-0.161			-0.694			-0.570**
			(0.439)			(0.307)			(0.537)			(0.226)
Constant	5.414***	5.341***	5.333***	5.533***	5.484***	5.477***	4.814***	4.809***	4.797***	4.683***	4.801***	4.779***
	(0.063)	(0.071)	(0.072)	(0.060)	(0.084)	(0.087)	(0.046)	(0.054)	(0.054)	(0.046)	(0.058)	(0.060)
Observations	207	207	207	225	225	225	207	207	207	225	225	225

* *p* < 0.10

** *p* < 0.05

*** *p* < 0.01

Since each BF personality trait is obtained as a mean over all questions about different aspects of a trait, estimates in Tables 5–7 may overlook one or more informative aspects of a trait if they cancel out when their mean is computed. Hence, in Tables H-L in S1 Appendix we also obtained estimates of regressions with as outcomes answers to questions used to construct BF traits, to gain a better understanding of the mechanisms underlying our results on BF traits. Estimates show that our result on the positive association between L and agreeableness is driven by significantly higher stated preferences for being considerate and kind, particularly for females, and for cooperation, particularly for males. As for the positive and weakly significant association between L and extraversion we found for females, it masks significantly higher preferences for being outgoing and little quiet.

Finally, in Table 8 we tested whether the relationship between earnings and personality differs by handedness. This was done by adding BF personality traits as independent variables to the earnings regressions we estimated in Table 4 and interacting them with the *L dummy*. Personality traits were normalised to quantify the change in earnings associated to the increase in a personality trait by one standard deviation. Estimates in Table 8 show that earnings, measured in euros, are not significantly different for L, with point estimates varying substantially (1-11% s.d.) and being negative in one specification. It also shows that earnings are significantly higher if CRT>0 (35-38% s.d.) and no significant gender difference is observed in these relationships.

**Table 8:** Regression of labour market payoffs (euros) in Ponti et al. (2016) [Unpublished] including Big Five personality traits.

L	0.554	0.462	0.133	-0.297	0.283	0.066
	(0.991)	(1.180)	(1.103)	(0.983)	(0.958)	(0.984)
F	0.320	0.300	0.232	0.301	0.194	0.242
	(0.477)	(0.477)	(0.478)	(0.480)	(0.476)	(0.479)
I(CRT>0)	1.722***	1.736***	1.741***	1.790***	1.880***	1.840***
	(0.516)	(0.519)	(0.518)	(0.517)	(0.516)	(0.522)
L*I(CRT>0)	0.158	0.276	0.784	1.443	0.125	0.295
	(1.706)	(1.862)	(1.601)	(1.445)	(1.634)	(1.635)
AGR		0.213	0.094	0.048	0.130	0.107
		(0.254)	(0.276)	(0.276)	(0.283)	(0.286)
L*AGR		-0.018	0.073	-0.014	-0.745	-0.327
		(1.040)	(0.973)	(0.943)	(0.834)	(0.876)
CON			0.353	0.316	0.285	0.241
			(0.266)	(0.267)	(0.266)	(0.279)
L*CON			1.324*	1.377**	1.694**	1.523*
			(0.750)	(0.698)	(0.785)	(0.866)
EXT				0.402	0.405	0.370
				(0.251)	(0.249)	(0.258)
L*EXT				1.001*	1.246**	1.360**
				(0.604)	(0.550)	(0.569)
NEU					0.380	0.374
					(0.271)	(0.272)
L*NEU					-1.896***	-2.025***
					(0.697)	(0.699)
OPE						0.166
						(0.273)
L*OPE						0.745
						(0.936)
Constant	5.549***	5.557***	5.588***	5.540***	5.557***	5.550***
	(0.406)	(0.406)	(0.406)	(0.408)	(0.407)	(0.409)
Observations	432	432	432	432	432	432

* *p* < 0.10

** *p* < 0.05

*** *p* < 0.01

The results in Table 8 are in line with those obtained in Table 4, thus suggesting that the personality traits added as extra independent variables are not highly correlated with the existing independent variables. Personality coefficients are not significant, suggesting that they are not relevant to explain earnings for right-handed. In contrast, earnings for L increase with conscientiousness (27-34% s.d.) although not all estimates are significant at the 5% level. Earnings also tend to significantly increase with extraversion (20-28% s.d.) and, finally, they significantly decrease with neuroticism (38-41% s.d.).

Results are unchanged when using standard errors clustered by session and independent subjects groups in a session, to correct them for that part of variation in payoffs that is due to subjects in the same session and group, as shown in Table E in S1 Appendix. Results separately by gender have not been obtained as including personality traits and their interaction with the L dummy in regressions by gender subsample leads to a substantial decrease in the power to test differences by handedness, given the small sample size and the low percentage of L in the population.

## Discussion

In this paper we tested experimentally whether left-handed (L) obtain significantly lower earnings (*H*_1_) by using data in Ponti et al. (2016) [Unpublished], that exploits a principal-agent setting to study labour market interactions in the lab. Differently from studies that found support for this hypothesis using survey data, we do not reject the null hypothesis of no difference in earnings. In addition, when we tested whether cognitive ability and personality are mechanisms explaining the variation in handedness and in earnings (*H*_2_), we found that only personality plays a significant role. In greater detail, we found a direct relationship between personality and earnings only for L with, for example, earnings increasing with extraversion, and an indirect positive relationship between being L, agreeableness and, for females, aspects of extraversion.

The novel relationship we found between earnings, L and personality traits, that are used as proxies for non-cognitive ability in the literature, suggests a potential role for personality aspects of non-cognitive ability to explain L’s behaviour. Our main contribution to the non-experimental literature, in addition to testing the relationship between handedness and earnings in a controlled environment, is highlighting the potential relevance of personality to interpret the existing evidence and to stimulate further research on its impact on decisions.

Our results have high internal validity as they are obtained using data from a laboratory experiment, i.e. a controlled environment, while, in contrast, they have low external validity as experimental subjects are not representative of the population. This suggests a possible reason why they differ with respect to those from studies using survey data and, moreover, that they are little suitable to formulate policy recommendations. For example, data on earnings are not representative across sectors in the real-life labour market. This notwithstanding, they were obtained in an experimental task which aims to take to the lab salient labour market features, i.e. agents are paid by principals to exert costly effort while principals claim residual profits once agents have been paid. In addition, differences in results are only partially due to differences in the CRT score distribution in our dataset relative to a larger and more representative one from a meta-analysis in [51], as the only non-negligible difference is in the percentage with 0 correct answers in the CRT.

Data on handedness are more closely in line with population figures. However, handedness is self-reported while a more precise measure accounting for consistency in hand-use across several activities, which has been shown to vary across subjects [5, 17, 38, 53], would be valuable to reduce measurement error. Finally, since we used a reduced version of the Big Five test not containing all questions, obtaining data on the missing questions may lead to changes in the estimates obtained from regressions with personality traits as outcomes. However, the evidence on higher preferences for cooperation and higher sociability for L is obtained using single questions that are used to construct personality traits and, hence, it does not depend on questions that were not asked to our experimental subjects.

We also contribute to the experimental literature studying the impact on decisions of cognitive and non-cognitive ability as the latter aspect of ability is proxied using data on personality in the literature. Our results highlight the importance of accounting for handedness, an easy-to-measure proxy of a predetermined characteristic associated with personality differences. Since handedness shows a low and non-significant correlation with gender and with cognitive ability, they may independently and jointly influence subjects’ decisions. In addition, the evidence on personality differences, that are significant for traits related to social interactions, suggests that accounting for L in experimental settings in which social interactions are salient may be more relevant than in settings in which they are not. However, a formal test of this conjecture is left for future research.

Additional questions related to the ones answered in this paper have been left for future research as a greater sample size and additional outcomes are required to answer them. In the experimental analysis of occupational choice, i.e. between a role as principal or agent, and of subjects’ earnings currently in progress in Ponti et al. (2016) [Unpublished], both exogenously varied institutions in the experiment, as well as handedness, ability, personality and gender may play a role. However, their relative importance is hard to predict ex-ante. In addition, studying the relationship between handedness and degree field of study may be valuable to assess whether the results are helpful to rationalise differences in occupational choice by handedness that are observed in survey data.

## Supporting Information

S1 Appendix Additional results on the relationship between earnings, handedness, ability and personality. (PDF)

S1 Data Dataset used in the empirical analysis. (XLS)

S2 Data Codebook containing names and labels of the variables in the dataset. (TXT)
