# Supplementary material for: Handedness, Earnings, Ability and Personality. Evidence from the Lab
Source: PLoS One. 2016 Oct 27;11(10):e0164412. doi: 10.1371/journal.pone.0164412 (PMC5082911; doi:10.1371/journal.pone.0164412)
Supplement: S1 Appendix — (PDF) [file pone.0164412.s001.pdf]

Handedness, Earnings, Ability and Personality. Evidence from the Lab.

S1 Appendix

Marcello Sartarelli*

Abstract

This Appendix shows in section A additional results on the relationship between earnings, handedness and ability. In addition, in section B it shows results also accounting for the role of personality by using data on personality traits in the Big Five test and on individual questions in the test.

Keywords: ability, Big Five, CRT, earnings, gender, handedness, left-handed, lefty, personality traits

JEL Classification: C91, D81, D87

*Universidad de Alicante, Departamento de Fundamentos del Análisis Económico, Alicante, Spain, Email: marcellosartarelli@gmail.com

A Handedness, Ability and Earnings

Table A is divided in two panels. Panel A shows on the left-hand side the number of subjects by handedness, ability and gender. The total number of left-handed (L) in the dataset is 35. L females with CRT>0 are 3, a low number that led to cautiously interpret regression estimates for this group in the paper. It also shows on the right-hand side the percentage of L and of subjects with CRT>0 by gender. Overall, they are similar to the percentage for the full data sample shown in Table 1 in the paper. In addition, females score worse in the CRT, in line with results in the related literature.

[Table A about HERE]

Panel B, in the bottom part of Table A, shows the distribution of the number of correct answers in the CRT for the full data sample and separately by handedness. The panel on the left-hand side shows that L are approximately 10% of the total number of subjects separately by CRT score. In addition, it shows that the number of L answering all questions correctly is equal to 2, i.e. very small.

Panel B in the same table also shows on the right-hand side that 60% of subjects does not answer any of the three questions in the CRT correctly, that 16.7% answers one correctly, 15% two and 7.6% all three. When we look at data separately by handedness, the distribution of right answers is similar although with L doing slightly worse. Finally, when we compare the distribution in our dataset with the one obtained using a large dataset in the meta-analysis in [1] we find that in our dataset the percentage of subjects with 0 right answers is higher by about 50%. For each of the remaining three categories of subjects by number of right answers, ranging from one to three, the difference is instead lower and varies between 10% and 20%.

Table B shows on the left-hand side OLS regressions with as outcome a dummy equal to 1 if CRT>0 and 0 otherwise. On the right-hand side it shows, instead, ordered logit estimates of the CRT score, taking integer values from 0 to 3, to account for the integer support of the CRT score. Estimates for the two different regressions show no significant difference in CRT by handedness while CRT is highly significantly lower for females.

[Table B about HERE]

Since survey evidence shows that labour market performance varies with cognitive and non-cognitive ability (see for a review [2]), we also borrowed from the Ponti et al. (2016) [Unpublished] dataset information on students' self-reported achievement at university (GPA). We use it as a joint measure of cognitive and non-cognitive ability as achievement at university requires a combination of cognitive ability, for example proficiency in English, math and science, as well as of non-cognitive ability, for example perseverance and punctuality. In the data sample used in the empirical analysis GPA ranges from 0 to 10, the mean value is about 6.8 and the standard deviation is close to unity.[1]

[Table C about HERE]

Table C shows regressions using as outcome students' GPA. Estimates on the left-hand side show that GPA is higher for L (13-39% s.d.) although the difference is weakly significant and the results is driven by females, as shown by subsample estimates by gender in the bottom part of the table. Estimates on the right-hand side, instead, have been obtained by also adding as explanatory variables dummies to absorb degree year effects since GPA was recorded at the time of the experiment, although their coefficients are not reported. A close comparison of estimates from regressions without and with degree year dummies shows that point estimates and their precision are very similar. In addition, the coefficient of the L dummy is more significant in some specifications when adding year dummies.

Since GPA is a proxy for cognitive and also non-cognitive ability, these results suggest that overall ability may be slightly higher for L, although the significance of the difference is not robust across specifications. When also taking into account that we found no significant differences in CRT by handedness, as shown in Table 3 in the paper, results on GPA seem to suggest that non-cognitive ability is weakly higher for L.[2]

[1]Positive and highly significant correlations in Table G between GPA and questions used to measure conscientiousness in the BF test, such as doing a thorough job and being perseverant, offer support to the claim that GPA measures cognitive and also non-cognitive ability.

[2]Studying the relationship between cognitive and non-cognitive ability, while potentially fruitful, is beyond the scope of this paper.

However, estimates of the regression of GPA on L must be interpreted with caution as self-reported information may be subject to measurement error. If the dependent variable suffers from measurement error, estimates of the relationship between GPA and L are upward biased.[3] This implies that the true value of the relationship between GPA and L is actually lower than the positive estimates shown in Table C.

[Table D about HERE]

To assess the precision of estimates of the regression of earnings on the L dummy in Table 4 in the paper, we re-estimated it by clustering standard errors jointly by experimental session and by independent group, in which subjects were randomly divided at the beginning of a session. This is to correct standard errors for that part of variation in earnings that is due to subjects in the same session and group. Estimates in Table D, with non-significant coefficients for the L dummy nor for its interaction with other variables, show that results are in line with the main results in Table 4 in the paper.

[Table E about HERE]

Similarly, Table E assesses estimates precision to clustering standard errors in augmented earnings regressions in which personality traits have been added as independent variables. Estimates are overall in line with the main results obtained in Table 6 in the paper. In greater details, they show that L's earnings are not significantly different while they are significantly higher if CRT>0. In addition, they show that for L earnings increase with conscientiousness although the difference is weakly significant, they tend to significantly increase with extraversion and, finally, that they significantly decrease with neuroticism. All personality traits used in the regressions have been normalised to facilitate the interpretation of estimates.

B Big Five Personality Traits and Questions

In addition to results on Big Five (BF) personality traits in the paper, in this section we also show summary statistics of the questions in the BF test, as they are used to

[3] This can be shown by adding an error term on the right-hand side of the regression of GPA on L and by substituting out for L.

obtain personality traits, correlations with our outcomes of interest and regressions using questions as outcomes.

[Table F about HERE]

Table F shows summary statistics of answers to the questions used to construct BF personality traits, that are added as explanatory variables in the empirical analysis in the paper. The support of BF questions are integers between 1 and 7, means of answers to questions vary approximately between 3 and 6 and the standard deviation varies approximately between 1 and 2. In addition, the low difference between mean and median values indicates that the distributions tend to be centered at the mean.[4]

[Table G about HERE]

Table G shows correlations between being L, GPA, labour market payoffs and BF questions. The first column on the left-hand side in the table shows that L is not significantly correlated with any variable except for a positive and weakly significant one with being considerate and kind (agreeableness). The second column shows that GPA is positively and weakly significantly correlated with preferences for cooperation (agreeableness) and highly significantly correlated with doing a thorough job, with being a reliable worker and with being perseverant (conscientiousness). It is also positively but weakly significantly correlated with being shy and with being full of energy (extraversion) while it is positively and highly significantly correlated with worrying a lot (neuroticism). GPA is also positively and highly significantly correlated with reflecting and playing with ideas, with being ingenious and significantly with having an active imagination and with being original and with new ideas (openness).

Finally, the third column in Table G shows that payoffs in the stylised labour market experiment in Ponti et al. (2016) [Unpublished] are negatively and highly significantly correlated with being generally trusting and with finding faults with others (agreeableness), with the tendency to be lazy (conscientiousness) and to be quiet (extraversion). They are also negatively but weakly significantly correlated

[4]See section 3 in the paper for additional details about the BF test.

with being reserved and with being shy (extraversion). In addition, they are positively and significantly correlated with reflecting and playing with ideas while they are negatively and highly significantly correlated with having few artistic interests (openness).

[Table H about HERE]

Table H shows estimates of regressions with as outcomes questions used to create the BF agreeableness (AGR) trait. Panel A shows on the left-hand side that the AGR trait is significantly higher for L and driven by females, as described in the paper in greater details. It also shows, on the right-hand side, that L quarrel less with others (13-17% s.d.) although the difference is not significant nor it is for any other explanatory variable.

Panel B in the same table shows on the left-hand side that L tend to be less trustful (1-23% s.d.). However, the difference is only significant once interactions with the CRT>0 dummy and the female one are accounted for, when the point estimate also increases (60% s.d.). In addition, L with CRT>0 tend to be significantly more trustful, with higher significance when accounting for the interactions with CRT and gender and it is driven by males, as shown by subsample estimates by gender.

On the right-hand side, Panel B in Table H shows that L have a higher tendency to find faults with others (4-32% s.d.) although the difference is small and not significant when not accounting for the interaction between L and the CRT dummies. When doing so, instead, the coefficient becomes negative and, when also accounting for interactions with the female dummy, it becomes significant (56% s.d.). In addition, L with CRT>0 are highly significantly more prone to find faults with others and this is driven by males, as shown by subsample estimates by gender.

Finally, Panel C in Table H shows on the left-hand side that L are significantly more considerate and kind (33-45% s.d.) and this is driven by females, as shown by subsample estimates by gender. On the right-hand side, it shows that preferences for cooperating are significantly higher for L (27-61% s.d.) and this tends to be driven by males, as shown by subsample estimates by gender.

[Table I about HERE]

Table I shows estimates using as outcomes questions used to create the BF conscientiousness (CON) trait. Panel A shows on the left-hand side the CON trait, that does not significantly vary with L nor with other explanatory variables, and on the right-hand side that the tendency to do a thorough job is higher for L (19-35% s.d.) although it is little significant across specifications.

Similarly, Panel B in the same table shows on the left-hand side that the tendency to be somewhat careless is higher for L (7-38% s.d.) although the difference is not significant, it is weakly significantly higher if CRT>0 (18-23% s.d.) and weakly significantly lower for L with CRT>0. On the right-hand side, Panel B shows that L are more reliable workers (7-16% s.d.) although the difference is not significant, similarly to coefficients of the remaining explanatory variables.

Finally, Panel C in Table I shows on that left-hand side that L are more perseverant (11-45% s.d.) although estimates are not significant. On the right-hand side, it shows that L are as lazy as others or tend to be less lazy (19-47% s.d.) and the difference is only significant when jointly accounting for interactions with CRT and gender dummies. When doing so, L are significantly less lazy and L with CRT>0 significantly lazier.

[Table J about HERE]

Table J shows estimates using as outcomes questions used to create the BF extraversion (EXT) trait. Panel A shows on the left-hand side that the EXT trait does not significantly vary with L although it is weakly significantly higher for L females, as described in the paper in greater details. The right-hand side shows that L tend to be less reserved (2-20% s.d.) although the difference is not significant nor are any other explanatory variables.

Panel B in the same table shows on the left-hand side that L are as shy as others, with estimates showing a mixed sign and not being significant. Similarly, the remaining explanatory variables are not significant. On the right-hand side, it shows that L tend to be significantly more outgoing (43-47% s.d.), once the interaction between L and CRT is taken into account, and this is driven by females, as shown by subsample estimates by gender.

Finally, Panel C in Table J shows on the left-hand side that L are more full of

energy (3-17% s.d.) although the difference is not significant, while it is significantly lower for females (22-35% s.d.). On the right-hand side it shows that L tend to be less quiet (3-38% s.d.) while L with CRT>0 more quiet, although coefficients are weakly significant and driven by females, as shown by subsample estimates by gender.

[Table K about HERE]

Table K shows estimates using as outcomes questions used to create the BF neuroticism (NEU) trait. Panel A shows on the left-hand side that the tendency to be relaxed is higher for L (8-21% s.d.) although the difference is not significant, it is highly significantly higher if CRT>0 (28-37% s.d.) and significantly lower for females (38-42% s.d.). Panel A also shows on the right-hand side that emotional stability is higher for L (3-18% s.d.) although the difference is not significant while it is highly significantly higher if CRT>0 (27% s.d.), although only when not controlling for gender. When controlling for it, instead, the CRT coefficient becomes smaller and loses significance and, in addition, the gender coefficient is negative and highly significant (39% s.d.). Finally, subsample estimates by gender show that emotional stability is significantly higher for L females with CRT>0. However, this estimate must be interpreted cautiously as only 3 subjects belong to this group.

Panel B in the same table shows on the left-hand side that L are less tense (2-11% s.d.) although the difference is not significant, nor are coefficients of the remaining independent variables. It also shows, on the right-hand side, that L worry more (2-36% s.d.) although the difference is not significant, L with CRT>0 worry less although estimates are weakly significant and, in addition, females worry highly significantly more (38% s.d.).

Finally, Panel C in Table K shows on the left-hand side that L are less moody (5-28% s.d.) although the difference is not significant. If CRT>0 the coefficient is negative (5-25% s.d.) and significant although not for all specifications while it is positive and highly significant for females (26-40% s.d.). Panel C also shows, on the right-hand side, that L's tendency to get nervous easily is significantly higher (27-57% s.d.). However, the significance level varies across specifications and it is driven by females, as shown by subsample estimates by gender. Conversely, it tends to be significantly lower for L with CRT>0, driven by males.

[Table L about HERE]

Table L shows estimates using as outcomes questions used to create the BF Openness (OPE) trait. Panel A shows on the left-hand side that L have higher preference for routine work (2-34% s.d.) although the difference is not significant while it is weakly significantly higher for L females, as shown by subsample estimates by gender. The panel also shows, on the right-hand side, that L reflect and play with ideas more (16-24% s.d.), although the difference is not significant. In addition it is significantly higher if CRT>0 (26-31% s.d.), although the significance level varies across specifications. It is also lower for females (20-21% s.d.) although not significant for all specifications.

Panel B in the same table shows on the left-hand side that being ingenious is higher for L (8-16% s.d.) although the difference is not significant. It is highly significantly higher if CRT>0 (20-35% s.d.) although it loses significance once the female dummy is fully interacted with the remaining explanatory variables. In addition, it is highly significantly lower for females (35-41% s.d.). Panel B also shows on the right-hand side that active imagination tends to be higher for L (17-18% s.d.) although not for all specifications and the coefficient is not significant. It is, instead, lower for females (14-29% s.d.) although the coefficient is not significant in all specifications.

Finally, Panel C in Table L shows on the left-hand side that L are more original (7-17% s.d.) although the difference is not significant, nor it is for the remaining explanatory variables. Similarly, the panel shows on the right-hand side that L are more curious (5-13% s.d.) although the difference is not significant, nor it is for the remaining explanatory variables.

References

[1] Brañas-Garza P, Kujal P, Lenkei B. Cognitive Reflection Test: Whom, How, When. University Library of Munich, Germany; 2015. 68049. Available from: https://mpra.ub.uni-muenchen.de/68049/1/MPRA_paper_68049.pdf.

[2] Heckman JJ, Kautz T. Hard Evidence on Soft Skills. Labour Economics. 2012;19(4):451–464. doi:10.1016/j.labeco.2012.05.014.

Table A: N. of observations and percentage by handedness, ability and gender

Panel A

	Number of observations				Percentage		
	L		R			L	CRT>0
	CRT=0	CRT>0	CRT=0	CRT>0			
F	14	3	136	54	F	0.082	0.275
M	8	10	103	104	M	0.080	0.507
Total	22	13	239	158	Overall	0.081	0.396

Panel B

N. CRT right	Number of observations			Percentage		
answers	Full sample	L	R	Full sample	L	R
0	261	22	239	0.604	0.629	0.602
1	73	7	66	0.169	0.200	0.166
2	65	4	61	0.150	0.114	0.154
3	33	2	31	0.076	0.057	0.078

Table B: Regressions of CRT score and of I(CRT>0)

	OLS regressions of I(CRT> 0)			Ologit regressions of CRT score		
L	-0.027	-0.025	0.053	-0.172	-0.212	-0.031
	(0.085)	(0.080)	(0.123)	(0.338)	(0.327)	(0.376)
F		-0.231***	-0.218***		-0.956***	-0.917***
		(0.046)	(0.048)		(0.200)	(0.207)
L*F			-0.161			-0.544
			(0.157)			(0.785)
Constant	0.398***	0.509***	0.502***			
	(0.025)	(0.034)	(0.035)			
Observations	432	432	432	432	432	432

* $p < 0.10$, ** $p < 0.05$, *** $p < 0.01$

Table C: Regressions of GPA without and with degree year dummies

Full sample regressions

	Without degree year dummies						With degree year dummies					
L	0.262*		0.266*	0.388*	0.384*	0.130	0.296**		0.298**	0.397*	0.395*	0.062
	(0.153)		(0.154)	(0.219)	(0.218)	(0.394)	(0.149)		(0.151)	(0.206)	(0.206)	(0.355)
I(CRT>0) (C)		0.144	0.146	0.172*	0.186*	0.172		0.128	0.129	0.151	0.156	0.129
		(0.089)	(0.089)	(0.094)	(0.095)	(0.127)		(0.089)	(0.089)	(0.094)	(0.096)	(0.129)
L*C				-0.328	-0.317	-0.014				-0.267	-0.264	0.186
				(0.283)	(0.284)	(0.456)				(0.282)	(0.283)	(0.427)
F					0.061	0.036					0.023	-0.013
					(0.088)	(0.114)					(0.088)	(0.115)
L*F						0.401						0.527
						(0.468)						(0.428)
F*C						0.025						0.051
						(0.192)						(0.192)
L*C*F						-0.615						-1.018*
						(0.547)						(0.539)
Constant	6.821***	6.785***	6.763***	6.753***	6.718***	6.732***	6.905***	6.899***	6.867***	6.858***	6.846***	6.865***
	(0.045)	(0.055)	(0.056)	(0.057)	(0.074)	(0.084)	(0.107)	(0.106)	(0.107)	(0.107)	(0.117)	(0.122)
Observations	432	432	432	432	432	432	432	432	432	432	432	432

Subsample regressions by gender

	Female			Male			Female			Male		
L	0.399*	0.416*	0.532**	0.132	0.123	0.130	0.399*	0.416*	0.532**	0.132	0.123	0.130
	(0.212)	(0.215)	(0.252)	(0.216)	(0.217)	(0.394)	(0.212)	(0.215)	(0.252)	(0.216)	(0.217)	(0.394)
C		0.159	0.196		0.171	0.172		0.159	0.196		0.171	0.172
		(0.137)	(0.144)		(0.122)	(0.127)		(0.137)	(0.144)		(0.122)	(0.127)
L*C			-0.630**			-0.014			-0.630**			-0.014
			(0.301)			(0.456)			(0.301)			(0.456)
Constant	6.824***	6.779***	6.768***	6.818***	6.733***	6.732***	6.824***	6.779***	6.768***	6.818***	6.733***	6.732***
	(0.065)	(0.076)	(0.077)	(0.064)	(0.083)	(0.084)	(0.065)	(0.076)	(0.077)	(0.064)	(0.083)	(0.084)
Observations	207	207	207	225	225	225	207	207	207	225	225	225

* $p < 0.10$, ** $p < 0.05$, *** $p < 0.01$

Table D: Regression of labour market payoffs (euros) in Ponti et al. (2016) [Unpublished] with standard errors clustered by experimental session and cohort

			Full sample regressions			
L	0.569		0.613	0.575	0.554	0.340
	(0.881)		(0.900)	(1.353)	(1.360)	(1.571)
I(CRT>0) (C)		1.652***	1.657***	1.649**	1.722**	2.662***
		(0.546)	(0.546)	(0.649)	(0.660)	(0.957)
L*C				0.101	0.158	-0.602
				(2.144)	(2.157)	(2.594)
F					0.320	1.068
					(0.426)	(0.641)
L*F						0.257
						(1.178)
F*C						-2.253**
						(1.090)
L*C*F						3.240
						(2.391)
Constant	6.388***	5.780***	5.728***	5.731***	5.549***	5.124***
	(0.818)	(0.808)	(0.818)	(0.833)	(0.864)	(0.887)
Observations	432	432	432	432	432	432

			Subsample regressions by gender			
		Female			Male	
L	1.018	1.079	0.597	0.147	0.008	0.340
	(1.207)	(1.207)	(1.400)	(1.102)	(1.070)	(1.569)
C		0.568	0.409		2.614***	2.662***
		(0.667)	(0.657)		(0.842)	(0.955)
L*C			2.638			-0.602
			(1.863)			(2.590)
Constant	6.308***	6.146***	6.191***	6.461***	5.148***	5.124***
	(0.839)	(0.888)	(0.886)	(0.860)	(0.857)	(0.885)
Observations	207	207	207	225	225	225

* $p < 0.10$, ** $p < 0.05$, *** $p < 0.01$

Table E: Regression of labour market payoffs (euros) in Ponti et al. (2016) [Unpublished] including BF traits with standard errors clustered by experimental session and cohort

L	0.554	0.462	0.133	-0.297	0.283	0.066
	(1.360)	(1.437)	(1.339)	(1.212)	(1.181)	(1.168)
Female	0.320	0.300	0.232	0.301	0.194	0.242
	(0.426)	(0.422)	(0.423)	(0.420)	(0.416)	(0.424)
I(CRT>0)	1.722**	1.736**	1.741**	1.790***	1.880***	1.840***
	(0.660)	(0.667)	(0.658)	(0.656)	(0.662)	(0.662)
L*I(CRT>0)	0.158	0.276	0.784	1.443	0.125	0.295
	(2.157)	(2.099)	(1.943)	(1.726)	(1.909)	(1.869)
BF AGR		0.213	0.094	0.048	0.130	0.107
		(0.298)	(0.318)	(0.318)	(0.311)	(0.303)
L*AGR		-0.018	0.073	-0.014	-0.745	-0.327
		(1.043)	(1.112)	(1.144)	(0.964)	(0.915)
BF CON			0.353	0.316	0.285	0.241
			(0.363)	(0.369)	(0.360)	(0.345)
L*CON			1.324	1.377*	1.694*	1.523*
			(0.801)	(0.737)	(0.869)	(0.901)
BF EXT				0.402*	0.405*	0.370
				(0.231)	(0.221)	(0.241)
L*EXT				1.001	1.246**	1.360**
				(0.662)	(0.584)	(0.599)
BF NEU					0.380	0.374
					(0.261)	(0.265)
L*NEU					-1.896***	-2.025***
					(0.652)	(0.618)
BF OPE						0.166
						(0.262)
L*OPE						0.745
						(0.789)
Constant	5.549***	5.557***	5.588***	5.540***	5.557***	5.550***
	(0.864)	(0.856)	(0.848)	(0.846)	(0.848)	(0.845)
Observations	432	432	432	432	432	432

* $p < 0.10$, ** $p < 0.05$, *** $p < 0.01$

Table F: Summary statistics of Big Five questions

	Mean	Median	Std. dev.	Min	Max	N. obs.s
	Agreeableness					
Quarrels with others	2.822	2.000	1.664	1.000	7.000	432
Generally trusting	2.583	2.000	2.595	1.000	7.000	432
Find faults with others	3.035	4.000	2.870	1.000	7.000	432
Considerate and kind	5.780	6.000	1.196	1.000	7.000	432
Likes to cooperate	5.755	6.000	1.220	1.000	7.000	432
	Conscientiousness					
Thorough job	5.444	6.000	1.356	1.000	7.000	432
Somewhat careless	4.294	5.000	1.681	1.000	7.000	432
Reliable worker	6.183	7.000	1.084	1.000	7.000	432
Perseverant	5.470	6.000	1.335	1.000	7.000	432
Tends to be lazy	1.713	1.000	1.963	1.000	7.000	432
	Extraversion					
Reserved	4.102	4.000	1.799	1.000	7.000	432
Sometimes shy, inhibited	3.944	4.000	1.818	1.000	7.000	432
Outgoing, sociable	5.294	6.000	1.470	1.000	7.000	432
Full of energy	5.493	6.000	1.269	1.000	7.000	432
Tends to be quiet	3.646	4.000	1.954	1.000	7.000	432
	Neuroticism					
Relaxed, handles stress well	4.407	4.500	1.843	1.000	7.000	432
Emotionally stable, not easily upset	4.502	5.000	1.688	1.000	7.000	432
Can be tense	3.525	4.000	1.706	1.000	7.000	432
Worries a lot	5.519	6.000	1.466	1.000	7.000	432
Moody	3.558	4.000	1.707	1.000	7.000	432
Gets nervous easily	3.896	4.000	1.741	1.000	7.000	432
	Openness					
Prefers routine work	3.127	3.000	1.646	1.000	7.000	432
Reflects and plays with ideas	5.512	6.000	1.350	1.000	7.000	432
Ingenious, deep thinker	5.338	5.500	1.247	1.000	7.000	432
Active imagination	5.356	6.000	1.311	1.000	7.000	432
Original, with new ideas	5.347	6.000	1.278	1.000	7.000	432
Curious about many things	5.706	6.000	1.258	1.000	7.000	432
Few artistic interests	1.759	1.000	2.026	1.000	7.000	432

Table G: Correlations between main outcomes and Big Five questions

	L	GPA	Exp. payoff
Left-handed (L)	1.000		
GPA	0.079	1.000	
Experiment payoff (euros)	0.031	0.023	1.000
Agreeableness			
Quarrels with others	-0.035	0.009	0.003
Generally trusting	-0.001	0.006	-0.187***
Find faults with others	0.011	0.025	-0.198***
Considerate and kind	0.090*	0.062	-0.050
Likes to cooperate	0.074	0.080*	0.077
Conscientiousness			
Thorough job	0.053	0.307***	-0.023
Somewhat careless	0.019	-0.044	0.014
Reliable worker	0.044	0.127***	0.015
Perseverant	0.029	0.265***	0.067
Tends to be lazy	0.005	-0.053	-0.184***
Extraversion			
Reserved	-0.022	0.074	-0.090*
Sometimes shy, inhibited	-0.000	0.081*	-0.080*
Outgoing, sociable	0.079	0.036	-0.003
Full of energy	0.045	0.083*	0.014
Tends to be quiet	-0.037	0.046	-0.137***
Neuroticism			
Relaxed, handles stress well	0.022	-0.054	-0.054
Emotionally stable, not easily upset	0.017	-0.000	-0.004
Can be tense	-0.027	0.060	0.044
Worries a lot	0.005	0.146***	0.002
Moody	-0.013	0.019	-0.026
Gets nervous easily	0.076	0.039	0.013
Openness			
Prefers routine work	0.075	-0.045	0.031
Reflects and play with ideas	0.045	0.156***	0.096**
Ingenious, deep thinker	0.022	0.150***	0.017
Active imagination	0.049	0.100**	0.044
Original, with new ideas	0.046	0.105**	-0.042
Curious about many things	0.036	0.078	-0.013
Few artistic interests	-0.002	-0.036	-0.229***

* $p < 0.10$, ** $p < 0.05$, *** $p < 0.01$

Table H: Big Five AGReeableness trait and questions

Panel A

	Full sample regressions											
	Agreeableness trait						Quarrels with others (AGR)					
L	0.161*		0.158*	0.311***	0.307***	0.309**	-0.210		-0.209	-0.275	-0.253	-0.240
	(0.091)		(0.089)	(0.099)	(0.099)	(0.130)	(0.279)		(0.279)	(0.306)	(0.305)	(0.555)
I(CRT>0) (C)		-0.090	-0.089	-0.057	-0.043	-0.062		0.063	0.061	0.047	-0.028	-0.029
		(0.064)	(0.064)	(0.068)	(0.070)	(0.091)		(0.165)	(0.166)	(0.173)	(0.176)	(0.243)
L*C				-0.408**	-0.397**	-0.508**				0.177	0.118	0.479
				(0.179)	(0.177)	(0.197)				(0.623)	(0.611)	(0.849)
F					0.063	0.037					-0.328**	-0.299
					(0.065)	(0.086)					(0.163)	(0.219)
L*F						-0.001						-0.022
						(0.189)						(0.662)
F*C						0.040						0.023
						(0.145)						(0.352)
L*C*F						0.478						-1.568
						(0.390)						(0.986)
Constant	4.839***	4.888***	4.875***	4.862***	4.826***	4.841***	2.839***	2.797***	2.815***	2.820***	3.007***	2.990***
	(0.033)	(0.040)	(0.041)	(0.042)	(0.057)	(0.066)	(0.084)	(0.101)	(0.106)	(0.107)	(0.145)	(0.170)
Observations	432	432	432	432	432	432	432	432	432	432	432	432

	Subsample regressions by gender											
	Female			Male			Female			Male		
L	0.305**	0.302**	0.308**	0.024	0.029	0.309**	-0.454	-0.462	-0.263	0.024	0.024	-0.240
	(0.125)	(0.126)	(0.138)	(0.121)	(0.115)	(0.130)	(0.318)	(0.317)	(0.361)	(0.435)	(0.435)	(0.554)
C		-0.024	-0.022		-0.102	-0.062		-0.071	-0.006		0.009	-0.029
		(0.108)	(0.113)		(0.085)	(0.091)		(0.243)	(0.255)		(0.232)	(0.242)
L*C			-0.030			-0.508**			-1.089**			0.479
			(0.337)			(0.197)			(0.502)			(0.849)
Constant	4.872***	4.878***	4.878***	4.810***	4.861***	4.841***	2.689***	2.710***	2.691***	2.976***	2.971***	2.990***
	(0.048)	(0.054)	(0.055)	(0.045)	(0.064)	(0.066)	(0.116)	(0.136)	(0.138)	(0.121)	(0.166)	(0.170)
Observations	207	207	207	225	225	225	207	207	207	225	225	225

* $p < 0.10$, ** $p < 0.05$, *** $p < 0.01$

Continues on the next page

Continues from the previous page

Panel B

	Full sample regressions											
	Generally trusting (AGR)						Find faults with others (AGR)					
L	-0.013		-0.020	-0.604	-0.581	-1.575**	0.118		0.118	-0.927	-0.912	-1.607**
	(0.436)		(0.441)	(0.570)	(0.580)	(0.663)	(0.504)		(0.504)	(0.565)	(0.570)	(0.766)
C		-0.269	-0.269	-0.392	-0.472*	-0.191		0.010	0.011	-0.210	-0.260	0.037
		(0.256)	(0.256)	(0.268)	(0.272)	(0.367)		(0.287)	(0.287)	(0.298)	(0.305)	(0.404)
L*C				1.564*	1.502*	2.641***				2.797***	2.759***	3.463***
				(0.842)	(0.840)	(0.979)				(0.963)	(0.965)	(1.160)
F					-0.348	-0.149					-0.218	0.003
					(0.253)	(0.340)					(0.283)	(0.369)
L*F						1.542						1.068
						(1.028)						(1.078)
F*C						-0.690						-0.722
						(0.546)						(0.617)
L*C*F						-2.403						-1.350
						(1.539)						(2.190)
Constant	2.584***	2.690***	2.691***	2.741***	2.939***	2.825***	3.025***	3.031***	3.021***	3.109***	3.233***	3.107***
	(0.131)	(0.160)	(0.164)	(0.168)	(0.221)	(0.255)	(0.144)	(0.173)	(0.180)	(0.182)	(0.243)	(0.278)
Observations	432	432	432	432	432	432	432	432	432	432	432	432
	Subsample regressions by gender											
	Female			Male			Female			Male		
L	0.103	0.010	-0.034	-0.118	-0.119	-1.575**	-0.092	-0.152	-0.539	0.319	0.302	-1.607**
	(0.664)	(0.662)	(0.786)	(0.572)	(0.572)	(0.663)	(0.701)	(0.718)	(0.759)	(0.718)	(0.699)	(0.766)
C		-0.866**	-0.880**		0.018	-0.191		-0.557	-0.684		0.311	0.037
		(0.385)	(0.404)		(0.348)	(0.367)		(0.454)	(0.467)		(0.386)	(0.404)
L*C			0.237			2.641***			2.113			3.463***
			(1.188)			(0.979)			(1.859)			(1.159)
Constant	2.426***	2.672***	2.676***	2.729***	2.720***	2.825***	2.916***	3.074***	3.110***	3.126***	2.969***	3.107***
	(0.188)	(0.220)	(0.224)	(0.183)	(0.249)	(0.255)	(0.208)	(0.241)	(0.243)	(0.201)	(0.272)	(0.278)
Observations	207	207	207	225	225	225	207	207	207	225	225	225

* $p < 0.10$, ** $p < 0.05$, *** $p < 0.01$

Continues on the next page

Continues from the previous page

Panel C

	Full sample regressions											
	Considerate and kind (AGR)						Likes to cooperate (AGR)					
L	0.395**		0.394**	0.528***	0.522***	0.532**	0.329**		0.327**	0.429**	0.427**	0.743***
	(0.174)		(0.174)	(0.178)	(0.178)	(0.266)	(0.153)		(0.152)	(0.195)	(0.196)	(0.277)
C		-0.023	-0.020	0.008	0.030	-0.007		-0.088	-0.085	-0.063	-0.058	-0.103
		(0.120)	(0.120)	(0.126)	(0.132)	(0.170)		(0.121)	(0.121)	(0.129)	(0.132)	(0.170)
L*C				-0.358	-0.341	-0.543				-0.272	-0.268	-0.697*
				(0.393)	(0.390)	(0.499)				(0.307)	(0.308)	(0.393)
F					0.093	0.046					0.022	-0.007
					(0.121)	(0.156)					(0.121)	(0.159)
L*F						-0.011						-0.493
						(0.355)						(0.375)
F*C						0.076						0.113
						(0.272)						(0.270)
L*C*F						0.855						1.021*
						(0.644)						(0.571)
Constant	5.748***	5.789***	5.756***	5.745***	5.692***	5.718***	5.728***	5.789***	5.762***	5.753***	5.741***	5.757***
	(0.061)	(0.072)	(0.075)	(0.076)	(0.106)	(0.122)	(0.063)	(0.074)	(0.077)	(0.079)	(0.101)	(0.115)
Observations	432	432	432	432	432	432	432	432	432	432	432	432
	Subsample regressions by gender											
	Female			Male			Female			Male		
L	0.569***	0.578***	0.521**	0.229	0.232	0.532**	0.306	0.309	0.250	0.350	0.359*	0.743***
	(0.206)	(0.204)	(0.236)	(0.269)	(0.267)	(0.266)	(0.217)	(0.217)	(0.253)	(0.218)	(0.211)	(0.277)
C		0.087	0.069		-0.050	-0.007		0.029	0.009		-0.159	-0.103
		(0.200)	(0.212)		(0.160)	(0.169)		(0.199)	(0.210)		(0.159)	(0.169)
L*C			0.312			-0.543			0.324			-0.697*
			(0.408)			(0.498)			(0.414)			(0.393)
Constant	5.784***	5.759***	5.765***	5.715***	5.740***	5.718***	5.753***	5.744***	5.750***	5.705***	5.785***	5.757***
	(0.088)	(0.096)	(0.098)	(0.084)	(0.118)	(0.122)	(0.093)	(0.108)	(0.110)	(0.084)	(0.111)	(0.115)
Observations	207	207	207	225	225	225	207	207	207	225	225	225

* $p < 0.10$, ** $p < 0.05$, *** $p < 0.01$

Table I: Big Five CONscientiousness trait and questions

Panel A

Full sample regressions

	CONscientiousness trait						Thorough job					
L	0.086		0.083	0.166	0.156	0.281	0.263		0.264	0.475*	0.464*	0.430
	(0.155)		(0.154)	(0.174)	(0.176)	(0.244)	(0.197)		(0.198)	(0.268)	(0.268)	(0.373)
I(CRT>0) (C)		-0.098	-0.097	-0.079	-0.044	-0.123		0.039	0.041	0.086	0.120	0.093
		(0.090)	(0.090)	(0.094)	(0.096)	(0.130)		(0.132)	(0.132)	(0.141)	(0.143)	(0.196)
L*C				-0.222	-0.194	-0.412				-0.565	-0.538	-0.543
				(0.338)	(0.335)	(0.422)				(0.371)	(0.369)	(0.490)
F					0.155*	0.085					0.150	0.121
					(0.089)	(0.118)					(0.133)	(0.183)
L*F						-0.189						0.058
						(0.339)						(0.522)
F*C						0.184						0.058
						(0.194)						(0.287)
L*C*F						0.646						0.130
						(0.615)						(0.692)
Constant	5.411***	5.457***	5.450***	5.443***	5.355***	5.394***	5.423***	5.429***	5.407***	5.389***	5.304***	5.320***
	(0.045)	(0.055)	(0.057)	(0.058)	(0.079)	(0.092)	(0.069)	(0.086)	(0.088)	(0.090)	(0.119)	(0.139)
Observations	432	432	432	432	432	432	432	432	432	432	432	432

Subsample by gender

	Female			Male			Female			Male		
L	0.127	0.135	0.092	0.045	0.054	0.281	0.398	0.412	0.487	0.133	0.130	0.430
	(0.206)	(0.206)	(0.236)	(0.227)	(0.223)	(0.243)	(0.304)	(0.306)	(0.365)	(0.247)	(0.249)	(0.372)
C		0.075	0.061		-0.156	-0.123		0.127	0.151		0.050	0.093
		(0.138)	(0.144)		(0.124)	(0.130)		(0.199)	(0.210)		(0.184)	(0.196)
L*C			0.234			-0.412			-0.413			-0.543
			(0.448)			(0.422)			(0.489)			(0.490)
Constant	5.497***	5.475***	5.479***	5.332***	5.411***	5.394***	5.484***	5.448***	5.441***	5.367***	5.342***	5.320***
	(0.063)	(0.073)	(0.074)	(0.065)	(0.090)	(0.092)	(0.098)	(0.117)	(0.119)	(0.098)	(0.135)	(0.139)
Observations	207	207	207	225	225	225	207	207	207	225	225	225

Continues on the next page

Continues from the previous page

Panel B

	Full sample regressions											
	Somewhat careless						Reliable worker					
L	0.115		0.124	0.507	0.526	0.632	0.174		0.169	0.171	0.158	0.075
	(0.282)		(0.288)	(0.344)	(0.342)	(0.542)	(0.161)		(0.161)	(0.192)	(0.190)	(0.315)
C		0.307*	0.308*	0.389**	0.325*	0.392*		-0.177	-0.175	-0.175	-0.131	-0.213
		(0.166)	(0.166)	(0.174)	(0.177)	(0.230)		(0.108)	(0.108)	(0.114)	(0.114)	(0.161)
L*C				-1.026*	-1.076*	-0.967				-0.003	0.032	0.063
				(0.583)	(0.574)	(0.780)				(0.344)	(0.340)	(0.474)
F					-0.281*	-0.199					0.194*	0.112
					(0.165)	(0.216)					(0.104)	(0.141)
L*F						-0.176						0.138
						(0.700)						(0.396)
F*C						-0.140						0.186
						(0.362)						(0.226)
L*C*F						-0.785						0.131
						(1.042)						(0.612)
Constant	4.285***	4.172***	4.162***	4.130***	4.290***	4.243***	6.169***	6.253***	6.239***	6.238***	6.128***	6.175***
	(0.085)	(0.102)	(0.106)	(0.107)	(0.139)	(0.158)	(0.055)	(0.065)	(0.068)	(0.069)	(0.092)	(0.108)
Observations	432	432	432	432	432	432	432	432	432	432	432	432

	Subsample by gender											
	Female			Male			Female			Male		
L	0.120	0.135	0.456	0.116	0.099	0.632	0.250	0.249	0.213	0.099	0.110	0.075
	(0.399)	(0.405)	(0.442)	(0.397)	(0.406)	(0.542)	(0.203)	(0.204)	(0.240)	(0.241)	(0.240)	(0.315)
C		0.147	0.252		0.315	0.392*		-0.016	-0.028		-0.208	-0.213
		(0.270)	(0.280)		(0.220)	(0.230)		(0.150)	(0.159)		(0.152)	(0.161)
L*C			-1.752**			-0.967			0.194			0.063
			(0.692)			(0.779)			(0.387)			(0.474)
Constant	4.116***	4.074***	4.044***	4.440***	4.281***	4.243***	6.279***	6.283***	6.287***	6.068***	6.172***	6.175***
	(0.125)	(0.145)	(0.147)	(0.115)	(0.155)	(0.158)	(0.074)	(0.089)	(0.091)	(0.080)	(0.105)	(0.108)
Observations	207	207	207	225	225	225	207	207	207	225	225	225

Continues on the next page

Continues from the previous page

Panel C

	Full sample regressions											
	Perseverant						Tends to be lazy					
L	0.142		0.144	0.317	0.314	0.602	0.033		0.036	-0.376	-0.369	-0.930**
	(0.229)		(0.230)	(0.317)	(0.317)	(0.381)	(0.335)		(0.333)	(0.376)	(0.379)	(0.429)
C		0.084	0.085	0.122	0.131	0.092		0.127	0.127	0.040	0.015	0.195
		(0.129)	(0.129)	(0.136)	(0.141)	(0.180)		(0.199)	(0.199)	(0.208)	(0.211)	(0.279)
L*C				-0.464	-0.457	-0.792				1.104	1.085	1.755**
				(0.435)	(0.434)	(0.532)				(0.699)	(0.698)	(0.836)
F					0.041	0.021					-0.108	0.026
					(0.134)	(0.179)					(0.191)	(0.243)
L*F						-0.450						0.867
						(0.586)						(0.675)
F*C						0.100						-0.438
						(0.290)						(0.428)
L*C*F						0.696						-1.488
						(0.771)						(1.256)
Constant	5.458***	5.437***	5.425***	5.410***	5.387***	5.398***	1.710***	1.663***	1.660***	1.695***	1.756***	1.680***
	(0.067)	(0.085)	(0.087)	(0.089)	(0.116)	(0.134)	(0.099)	(0.114)	(0.119)	(0.121)	(0.161)	(0.181)
Observations	432	432	432	432	432	432	432	432	432	432	432	432

	Subsample by gender											
	Female			Male			Female			Male		
L	0.115	0.135	0.152	0.167	0.165	0.602	0.010	-0.014	-0.063	0.056	0.038	-0.930**
	(0.369)	(0.368)	(0.446)	(0.278)	(0.280)	(0.381)	(0.448)	(0.449)	(0.522)	(0.495)	(0.479)	(0.429)
C		0.186	0.192		0.030	0.092		-0.227	-0.243		0.334	0.195
		(0.215)	(0.227)		(0.171)	(0.180)		(0.309)	(0.325)		(0.266)	(0.279)
L*C			-0.097			-0.792			0.267			1.755**
			(0.558)			(0.532)			(0.938)			(0.836)
Constant	5.474***	5.421***	5.419***	5.444***	5.430***	5.398***	1.637***	1.701***	1.706***	1.778***	1.610***	1.680***
	(0.101)	(0.118)	(0.120)	(0.090)	(0.130)	(0.134)	(0.141)	(0.161)	(0.163)	(0.139)	(0.177)	(0.181)
Observations	207	207	207	225	225	225	207	207	207	225	225	225

* $p < 0.10$, ** $p < 0.05$, *** $p < 0.01$

Table J: Big Five EXTraversion trait and questions

Panel A

	Full sample regressions											
	EXTraversion trait						Reserved (EXT)					
L	0.209		0.205	0.420	0.434	0.046	-0.142		-0.137	-0.331	-0.353	0.030
	(0.224)		(0.221)	(0.279)	(0.283)	(0.472)	(0.318)		(0.315)	(0.431)	(0.434)	(0.650)
I(CRT>0) (C)		-0.175	-0.173	-0.127	-0.176	-0.258		0.180	0.179	0.138	0.212	0.251
		(0.127)	(0.128)	(0.133)	(0.135)	(0.174)		(0.176)	(0.176)	(0.185)	(0.188)	(0.237)
L*C				-0.576	-0.614	-0.217				0.520	0.578	0.074
				(0.443)	(0.446)	(0.638)				(0.606)	(0.604)	(0.832)
F					-0.213^{*}	-0.310^{*}					0.326^{*}	0.376
					(0.125)	(0.160)					(0.178)	(0.231)
L*F						0.621						-0.608
						(0.585)						(0.864)
F*C						0.176						-0.083
						(0.275)						(0.388)
L*C*F						-0.620						1.115
						(0.862)						(1.145)
Constant	4.602^{***}	4.688^{***}	4.671^{***}	4.653^{***}	4.774^{***}	4.829^{***}	4.113^{***}	4.031^{***}	4.042^{***}	4.059^{***}	3.873^{***}	3.845^{***}
	(0.064)	(0.078)	(0.080)	(0.081)	(0.102)	(0.114)	(0.090)	(0.112)	(0.115)	(0.117)	(0.146)	(0.164)
Observations	432	432	432	432	432	432	432	432	432	432	432	432

	Subsample regressions by gender											
	Female			Male			Female			Male		
L	0.528^{*}	0.513^{*}	0.667^{*}	-0.088	-0.074	0.046	-0.386	-0.360	-0.578	0.085	0.071	0.030
	(0.306)	(0.303)	(0.345)	(0.320)	(0.317)	(0.472)	(0.492)	(0.487)	(0.569)	(0.408)	(0.408)	(0.650)
C		-0.132	-0.082		-0.275	-0.258		0.240	0.168		0.257	0.251
		(0.204)	(0.213)		(0.167)	(0.174)		(0.293)	(0.307)		(0.227)	(0.237)
L*C			-0.837			-0.217			1.189			0.074
			(0.580)			(0.637)			(0.786)			(0.832)
Constant	4.496^{***}	4.533^{***}	4.519^{***}	4.700^{***}	4.838^{***}	4.829^{***}	4.268^{***}	4.200^{***}	4.221^{***}	3.971^{***}	3.842^{***}	3.845^{***}
	(0.095)	(0.111)	(0.113)	(0.087)	(0.112)	(0.114)	(0.138)	(0.161)	(0.163)	(0.118)	(0.160)	(0.164)
Observations	207	207	207	225	225	225	207	207	207	225	225	225

* $p < 0.10$, ** $p < 0.05$, *** $p < 0.01$

Continues on the next page

Continues from the previous page

Panel B

	Full sample regressions											
	Sometimes shy, inhibited (EXT)						Outgoing, sociable (EXT)					
L	-0.002		0.006	-0.316	-0.334	0.475	0.426		0.421	0.686***	0.699***	0.640*
	(0.295)		(0.289)	(0.392)	(0.400)	(0.603)	(0.263)		(0.260)	(0.250)	(0.250)	(0.354)
C		0.276	0.276	0.208	0.267	0.330		-0.196	-0.193	-0.137	-0.178	-0.303
		(0.181)	(0.181)	(0.192)	(0.196)	(0.260)		(0.148)	(0.148)	(0.153)	(0.153)	(0.199)
L*C				0.862	0.909*	0.145				-0.710	-0.742	-0.722
				(0.540)	(0.546)	(0.753)				(0.592)	(0.593)	(0.752)
F					0.261	0.372					-0.184	-0.302*
					(0.180)	(0.232)					(0.140)	(0.181)
L*F						-1.282						0.105
						(0.779)						(0.491)
F*C						-0.112						0.286
						(0.399)						(0.311)
L*C*F						1.089						0.144
						(1.087)						(1.118)
Constant	3.945***	3.835***	3.835***	3.862***	3.713***	3.650***	5.259***	5.372***	5.336***	5.314***	5.419***	5.485***
	(0.092)	(0.109)	(0.112)	(0.114)	(0.155)	(0.177)	(0.074)	(0.087)	(0.091)	(0.092)	(0.114)	(0.126)
Observations	432	432	432	432	432	432	432	432	432	432	432	432

	Subsample regressions by gender											
	Female			Male			Female			Male		
L	-0.614	-0.582	-0.808	0.572	0.554	0.475	0.645**	0.639**	0.745**	0.222	0.241	0.640*
	(0.439)	(0.431)	(0.493)	(0.369)	(0.367)	(0.603)	(0.312)	(0.314)	(0.340)	(0.416)	(0.406)	(0.354)
C		0.293	0.219		0.342	0.330		-0.052	-0.017		-0.360*	-0.303
		(0.289)	(0.302)		(0.246)	(0.260)		(0.229)	(0.238)		(0.192)	(0.199)
L*C			1.234			0.145			-0.578			-0.722
			(0.784)			(0.753)			(0.827)			(0.752)
Constant	4.084***	4.001***	4.022***	3.816***	3.645***	3.650***	5.179***	5.194***	5.184***	5.333***	5.514***	5.485***
	(0.130)	(0.147)	(0.149)	(0.130)	(0.172)	(0.177)	(0.109)	(0.129)	(0.130)	(0.100)	(0.125)	(0.126)
Observations	207	207	207	225	225	225	207	207	207	225	225	225

* $p < 0.10$, ** $p < 0.05$, *** $p < 0.01$

Continues on the next page

Continues from the previous page

Panel C

	Full sample regressions											
	Full of energy (EXT)						Tends to be quiet (EXT)					
L	0.210		0.211	0.036	0.054	0.032	-0.268		-0.260	-0.731*	-0.732*	-0.063
	(0.200)		(0.199)	(0.269)	(0.267)	(0.480)	(0.344)		(0.337)	(0.413)	(0.415)	(0.759)
C		0.065	0.066	0.029	-0.033	-0.218		0.286	0.284	0.184	0.189	0.187
		(0.125)	(0.125)	(0.132)	(0.136)	(0.175)		(0.194)	(0.194)	(0.203)	(0.207)	(0.273)
L*C				0.471	0.422	0.368				1.260*	1.263*	0.513
				(0.378)	(0.383)	(0.562)				(0.663)	(0.666)	(0.999)
F					-0.274**	-0.446***					0.021	0.055
					(0.127)	(0.164)					(0.192)	(0.250)
L*F						0.054						-1.055
						(0.576)						(0.888)
F*C						0.428						0.029
						(0.279)						(0.420)
L*C*F						0.398						1.438
						(0.869)						(1.260)
Constant	5.476***	5.467***	5.450***	5.464***	5.620***	5.718***	3.668***	3.533***	3.554***	3.594***	3.582***	3.563***
	(0.064)	(0.078)	(0.081)	(0.082)	(0.108)	(0.123)	(0.098)	(0.119)	(0.122)	(0.124)	(0.163)	(0.185)
Observations	432	432	432	432	432	432	432	432	432	432	432	432

	Subsample regressions by gender											
	Female			Male			Female			Male		
L	0.198	0.225	0.085	0.225	0.235	0.032	-0.797*	-0.761*	-1.118**	0.232	0.220	-0.063
	(0.294)	(0.289)	(0.318)	(0.267)	(0.271)	(0.479)	(0.441)	(0.430)	(0.461)	(0.498)	(0.496)	(0.758)
C		0.255	0.209		-0.189	-0.218		0.333	0.216		0.227	0.187
		(0.209)	(0.218)		(0.166)	(0.175)		(0.307)	(0.320)		(0.262)	(0.273)
L*C			0.767			0.368			1.951**			0.513
			(0.663)			(0.562)			(0.767)			(0.999)
Constant	5.332***	5.259***	5.272***	5.609***	5.704***	5.718***	3.679***	3.584***	3.618***	3.657***	3.543***	3.563***
	(0.094)	(0.107)	(0.109)	(0.087)	(0.120)	(0.123)	(0.142)	(0.166)	(0.168)	(0.136)	(0.182)	(0.185)
Observations	207	207	207	225	225	225	207	207	207	225	225	225

* $p < 0.10$, ** $p < 0.05$, *** $p < 0.01$

Table K: Big Five NEUroticism trait and questions

Panel A

	Full sample regressions											
	Relaxed, handles stress well						Emotionally stable, not easily upset					
L	0.147		0.165	0.333	0.385	0.610	0.106		0.119	0.054	0.098	0.311
	(0.325)		(0.327)	(0.434)	(0.421)	(0.452)	(0.296)		(0.292)	(0.395)	(0.383)	(0.530)
I(CRT>0) (C)		0.652***	0.653***	0.689***	0.513***	0.601**		0.475***	0.476***	0.463***	0.315*	0.320
		(0.175)	(0.176)	(0.183)	(0.182)	(0.241)		(0.164)	(0.164)	(0.172)	(0.172)	(0.227)
L*C				-0.451	-0.589	-0.926				0.174	0.058	-0.620
				(0.644)	(0.633)	(0.701)				(0.573)	(0.590)	(0.715)
F					-0.775***	-0.691***					-0.649***	-0.667***
					(0.175)	(0.240)					(0.163)	(0.217)
L*F						-0.363						-0.333
						(0.761)						(0.742)
F*C						-0.202						-0.028
						(0.371)						(0.350)
L*C*F						0.789						2.328**
						(1.379)						(1.029)
Constant	4.395***	4.149***	4.136***	4.121***	4.562***	4.515***	4.494***	4.314***	4.304***	4.310***	4.679***	4.689***
	(0.093)	(0.116)	(0.119)	(0.121)	(0.152)	(0.176)	(0.085)	(0.105)	(0.107)	(0.109)	(0.141)	(0.162)
Observations	432	432	432	432	432	432	432	432	432	432	432	432
	Subsample by gender											
	Female			Male			Female			Male		
L	0.181	0.223	0.248	0.128	0.100	0.610	0.248	0.290	-0.022	-0.017	-0.031	0.311
	(0.532)	(0.533)	(0.612)	(0.360)	(0.372)	(0.452)	(0.473)	(0.461)	(0.520)	(0.356)	(0.361)	(0.530)
C		0.390	0.399		0.528**	0.601**		0.395	0.293		0.271	0.320
		(0.273)	(0.282)		(0.228)	(0.241)		(0.258)	(0.267)		(0.216)	(0.227)
L*C			-0.137			-0.926			1.707**			-0.620
			(1.187)			(0.701)			(0.740)			(0.715)
Constant	3.937***	3.826***	3.824***	4.816***	4.551***	4.515***	4.105***	3.993***	4.022***	4.850***	4.714***	4.689***
	(0.134)	(0.161)	(0.163)	(0.122)	(0.171)	(0.176)	(0.121)	(0.143)	(0.145)	(0.114)	(0.157)	(0.162)
Observations	207	207	207	225	225	225	207	207	207	225	225	225

* $p < 0.10$, ** $p < 0.05$, *** $p < 0.01$

Continues on the next page

Continues from the previous page

Panel B

Full sample regressions

	Can be tense						Worries a lot					
L	-0.168		-0.174	-0.024	-0.047	-0.187	0.026		0.023	0.374	0.336	0.527
	(0.330)		(0.328)	(0.426)	(0.423)	(0.606)	(0.255)		(0.253)	(0.274)	(0.274)	(0.454)
C		-0.221	-0.223	-0.191	-0.115	-0.148		-0.123	-0.122	-0.048	0.078	0.084
		(0.168)	(0.168)	(0.174)	(0.177)	(0.229)		(0.146)	(0.147)	(0.153)	(0.154)	(0.217)
L*C				-0.400	-0.340	-0.002				-0.938*	-0.839	-1.334*
				(0.662)	(0.665)	(0.842)				(0.535)	(0.512)	(0.693)
F					0.337**	0.313					0.555***	0.549***
					(0.168)	(0.216)					(0.141)	(0.190)
L*F						0.223						-0.299
						(0.832)						(0.568)
F*C						0.084						-0.023
						(0.362)						(0.306)
L*C*F						-1.053						1.606*
						(1.440)						(0.840)
Constant	3.539***	3.613***	3.628***	3.615***	3.424***	3.437***	5.516***	5.567***	5.565***	5.536***	5.220***	5.223***
	(0.085)	(0.105)	(0.107)	(0.108)	(0.140)	(0.157)	(0.074)	(0.088)	(0.092)	(0.093)	(0.129)	(0.152)
Observations	432	432	432	432	432	432	432	432	432	432	432	432

Subsample by gender

	Female			Male			Female			Male		
L	-0.143	-0.157	0.036	-0.196	-0.188	-0.187	0.269	0.278	0.228	-0.210	-0.209	0.527
	(0.511)	(0.510)	(0.571)	(0.420)	(0.421)	(0.606)	(0.287)	(0.286)	(0.342)	(0.381)	(0.381)	(0.454)
C		-0.128	-0.065		-0.149	-0.148		0.078	0.061		-0.021	0.084
		(0.273)	(0.280)		(0.220)	(0.229)		(0.205)	(0.217)		(0.207)	(0.217)
L*C			-1.054			-0.002			0.272			-1.334*
			(1.168)			(0.842)			(0.476)			(0.692)
Constant	3.732***	3.768***	3.750***	3.362***	3.437***	3.437***	5.789***	5.767***	5.772***	5.266***	5.276***	5.223***
	(0.126)	(0.147)	(0.149)	(0.114)	(0.154)	(0.157)	(0.096)	(0.112)	(0.114)	(0.108)	(0.148)	(0.152)
Observations	207	207	207	225	225	225	207	207	207	225	225	225

* $p < 0.10$, ** $p < 0.05$, *** $p < 0.01$

Continues on the next page

Continues from the previous page

Panel C

	Full sample regressions											
	Moody						Gets nervous easily					
L	-0.079		-0.090	-0.100	-0.130	-0.475	0.486		0.477*	0.996***	0.960***	0.964*
	(0.308)		(0.306)	(0.395)	(0.383)	(0.541)	(0.297)		(0.285)	(0.299)	(0.299)	(0.525)
C		-0.430**	-0.430**	-0.433**	-0.330*	-0.080		-0.370**	-0.366**	-0.256	-0.131	-0.402*
		(0.166)	(0.166)	(0.173)	(0.177)	(0.224)		(0.172)	(0.172)	(0.180)	(0.182)	(0.238)
L*C				0.027	0.108	0.805				-1.391**	-1.294**	-1.348*
				(0.625)	(0.637)	(0.780)				(0.584)	(0.571)	(0.740)
F					0.451***	0.680***					0.546***	0.302
					(0.167)	(0.216)					(0.171)	(0.225)
L*F						0.517						0.020
						(0.741)						(0.637)
F*C						-0.579						0.628*
						(0.363)						(0.369)
L*C*F						-2.218**						0.384
						(1.072)						(1.394)
Constant	3.564***	3.728***	3.736***	3.736***	3.480***	3.350***	3.856***	4.042***	4.002***	3.958***	3.647***	3.786***
	(0.086)	(0.106)	(0.108)	(0.110)	(0.139)	(0.155)	(0.087)	(0.106)	(0.110)	(0.111)	(0.148)	(0.170)
Observations	432	432	432	432	432	432	432	432	432	432	432	432

	Subsample by gender											
	Female			Male			Female			Male		
L	-0.136	-0.216	0.042	-0.031	-0.031	-0.475	0.789**	0.807**	0.983***	0.193	0.220	0.964*
	(0.467)	(0.443)	(0.506)	(0.401)	(0.402)	(0.541)	(0.362)	(0.370)	(0.362)	(0.424)	(0.399)	(0.525)
C		-0.744***	-0.659**		-0.017	-0.080		0.169	0.227		-0.508**	-0.402*
		(0.274)	(0.286)		(0.214)	(0.224)		(0.275)	(0.283)		(0.227)	(0.238)
L*C			-1.412*			0.805			-0.965			-1.348*
			(0.736)			(0.779)			(1.182)			(0.740)
Constant	3.842***	4.054***	4.029***	3.309***	3.318***	3.350***	4.153***	4.105***	4.088***	3.585***	3.840***	3.786***
	(0.129)	(0.148)	(0.150)	(0.111)	(0.152)	(0.155)	(0.126)	(0.147)	(0.148)	(0.119)	(0.165)	(0.169)
Observations	207	207	207	225	225	225	207	207	207	225	225	225

* $p < 0.10$, ** $p < 0.05$, *** $p < 0.01$

Table L: Big Five OPEnness trait and questions

Panel A

Full sample regressions

	Prefers routine work						Reflects and plays with ideas					
L	0.452		0.450	0.565	0.566	0.036	0.221		0.231	0.310	0.328	0.265
	(0.302)		(0.301)	(0.388)	(0.389)	(0.609)	(0.179)		(0.179)	(0.220)	(0.220)	(0.372)
I(CRT>0) (C)		-0.095	-0.091	-0.067	-0.071	-0.185		0.402***	0.404***	0.420***	0.360**	0.351*
		(0.161)	(0.161)	(0.168)	(0.173)	(0.232)		(0.128)	(0.128)	(0.136)	(0.139)	(0.183)
L*C				-0.308	-0.311	0.035				-0.211	-0.258	-0.201
				(0.612)	(0.616)	(0.767)				(0.374)	(0.373)	(0.533)
F					-0.021	-0.169					-0.267**	-0.280
					(0.166)	(0.218)					(0.133)	(0.187)
L*F						0.848						0.101
						(0.783)						(0.462)
F*C						0.233						0.017
						(0.351)						(0.285)
L*C*F						-0.012						-0.072
						(1.570)						(0.683)
Constant	3.091***	3.165***	3.127***	3.117***	3.129***	3.214***	5.494***	5.352***	5.333***	5.326***	5.479***	5.485***
	(0.082)	(0.103)	(0.105)	(0.107)	(0.146)	(0.168)	(0.069)	(0.087)	(0.091)	(0.093)	(0.118)	(0.138)
Observations	432	432	432	432	432	432	432	432	432	432	432	432

Subsample by gender

	Female			Male			Female			Male		
L	0.883*	0.889*	0.884*	0.046	0.056	0.036	0.278	0.316	0.366	0.171	0.154	0.265
	(0.461)	(0.464)	(0.492)	(0.373)	(0.374)	(0.609)	(0.230)	(0.233)	(0.274)	(0.269)	(0.269)	(0.372)
C		0.050	0.048		-0.182	-0.185		0.352*	0.368*		0.335*	0.351*
		(0.260)	(0.264)		(0.221)	(0.232)		(0.206)	(0.218)		(0.173)	(0.183)
L*C			0.023			0.035			-0.273			-0.201
			(1.371)			(0.767)			(0.427)			(0.533)
Constant	3.058***	3.044***	3.044***	3.121***	3.212***	3.214***	5.311***	5.211***	5.206***	5.662***	5.493***	5.485***
	(0.117)	(0.138)	(0.138)	(0.115)	(0.164)	(0.168)	(0.103)	(0.123)	(0.125)	(0.092)	(0.134)	(0.138)
Observations	207	207	207	225	225	225	207	207	207	225	225	225

* $p < 0.10$, ** $p < 0.05$, *** $p < 0.01$

Continues on the next page

Continues from the previous page

Panel B

	Full sample regressions											
	Ingenious, a deep thinker						Active imagination					
L	0.099		0.110	0.163	0.193	0.178	0.234		0.238	0.220	0.232	-0.120
	(0.193)		(0.192)	(0.250)	(0.245)	(0.371)	(0.229)		(0.228)	(0.306)	(0.308)	(0.451)
C		0.428***	0.429***	0.440***	0.341***	0.246		0.146	0.148	0.144	0.101	-0.111
		(0.120)	(0.120)	(0.126)	(0.130)	(0.151)		(0.129)	(0.129)	(0.135)	(0.139)	(0.177)
L*C				-0.143	-0.221	-0.071				0.049	0.015	0.636
				(0.387)	(0.376)	(0.453)				(0.453)	(0.451)	(0.558)
F					-0.438***	-0.513***					-0.188	-0.377**
					(0.125)	(0.160)					(0.131)	(0.167)
L*F						0.031						0.574
						(0.493)						(0.608)
F*C						0.228						0.493*
						(0.275)						(0.283)
L*C*F						-0.546						-1.589
						(1.015)						(1.092)
Constant	5.330***	5.169***	5.159***	5.155***	5.404***	5.447***	5.338***	5.299***	5.279***	5.280***	5.387***	5.495***
	(0.063)	(0.078)	(0.081)	(0.083)	(0.101)	(0.112)	(0.066)	(0.081)	(0.083)	(0.084)	(0.109)	(0.122)
Observations	432	432	432	432	432	432	432	432	432	432	432	432
	Subsample by gender											
	Female			Male			Female			Male		
L	0.049	0.096	0.209	0.152	0.139	0.178	0.244	0.279	0.454	0.227	0.230	-0.120
	(0.305)	(0.312)	(0.325)	(0.219)	(0.219)	(0.371)	(0.369)	(0.376)	(0.408)	(0.276)	(0.279)	(0.451)
C		0.437*	0.474**		0.240*	0.246		0.325	0.382*		-0.060	-0.111
		(0.222)	(0.230)		(0.143)	(0.151)		(0.215)	(0.221)		(0.168)	(0.177)
L*C			-0.616			-0.071			-0.954			0.636
			(0.908)			(0.453)			(0.939)			(0.558)
Constant	5.068***	4.944***	4.934***	5.570***	5.449***	5.447***	5.226***	5.134***	5.118***	5.440***	5.470***	5.495***
	(0.101)	(0.114)	(0.115)	(0.076)	(0.108)	(0.111)	(0.098)	(0.114)	(0.115)	(0.088)	(0.119)	(0.121)
Observations	207	207	207	225	225	225	207	207	207	225	225	225

* $p < 0.10$, ** $p < 0.05$, *** $p < 0.01$

Continues on the next page

Continues from the previous page

Panel C

Full sample regressions

	Original, with new ideas						Curious about many things					
L	0.213		0.217	0.187	0.201	0.083	0.164		0.167	0.157	0.153	0.061
	(0.185)		(0.185)	(0.228)	(0.228)	(0.380)	(0.224)		(0.224)	(0.318)	(0.318)	(0.447)
C		0.161	0.163	0.156	0.110	0.034		0.080	0.081	0.079	0.093	-0.026
		(0.126)	(0.126)	(0.133)	(0.140)	(0.175)		(0.121)	(0.121)	(0.127)	(0.130)	(0.178)
L*C				0.081	0.045	0.366				0.025	0.036	0.176
				(0.388)	(0.384)	(0.478)				(0.419)	(0.419)	(0.571)
F					-0.204	-0.263					0.058	-0.050
					(0.131)	(0.170)					(0.123)	(0.170)
L*F						0.192						0.157
						(0.476)						(0.622)
F*C						0.182						0.275
						(0.290)						(0.260)
L*C*F						-1.010						-0.282
						(1.015)						(0.733)
Constant	5.330***	5.284***	5.265***	5.268***	5.384***	5.417***	5.693***	5.674***	5.660***	5.661***	5.628***	5.689***
	(0.065)	(0.079)	(0.082)	(0.084)	(0.114)	(0.130)	(0.063)	(0.081)	(0.082)	(0.084)	(0.110)	(0.129)
Observations	432	432	432	432	432	432	432	432	432	432	432	432

Subsample by gender

	Female			Male			Female			Male		
L	0.137	0.156	0.274	0.287	0.284	0.083	0.172	0.198	0.217	0.157	0.158	0.061
	(0.281)	(0.286)	(0.286)	(0.237)	(0.236)	(0.380)	(0.355)	(0.354)	(0.433)	(0.279)	(0.280)	(0.447)
C		0.177	0.216		0.063	0.034		0.243	0.249		-0.012	-0.026
		(0.224)	(0.231)		(0.165)	(0.175)		(0.179)	(0.189)		(0.169)	(0.178)
L*C			-0.645			0.366			-0.106			0.176
			(0.896)			(0.478)			(0.460)			(0.571)
Constant	5.216***	5.165***	5.154***	5.435***	5.403***	5.417***	5.711***	5.642***	5.640***	5.676***	5.682***	5.689***
	(0.097)	(0.109)	(0.110)	(0.087)	(0.126)	(0.130)	(0.090)	(0.109)	(0.110)	(0.089)	(0.126)	(0.129)
Observations	207	207	207	225	225	225	207	207	207	225	225	225

* $p < 0.10$, ** $p < 0.05$, *** $p < 0.01$
